# *In silico* Identification of Immune Cell-Types and Pathways Involved in Chronic Spontaneous Urticaria

**DOI:** 10.3389/fmed.2022.926753

**Published:** 2022-07-07

**Authors:** Connor Prosty, Sofianne Gabrielli, Moshe Ben-Shoshan, Michelle Le, Ana M. Giménez-Arnau, Ivan V. Litvinov, Philippe Lefrançois, Elena Netchiporouk

**Affiliations:** ^1^Faculty of Medicine, McGill University, Montreal, QC, Canada; ^2^Division of Allergy, Immunology and Dermatology, Montreal Children’s Hospital, Montreal, QC, Canada; ^3^Division of Dermatology, McGill University, Montreal, QC, Canada; ^4^Department of Dermatology, Hospital del Mar, Institut Mar d’Investigacions Mediques (IMIM), Universitat Autònoma de Barcelona (UAB), Barcelona, Spain; ^5^Division of Dermatology, University of Ottawa, Ottawa, ON, Canada

**Keywords:** chronic spontaneous urticaria, chronic urticaria, autoimmunity, autoallergy, chronic idiopathic urticaria, pathogenesis, Th2, Th17

## Abstract

**Background:**

The immunopathogenesis of chronic spontaneous urticaria (CSU) is poorly understood, but recent research suggests that patients can be divided into autoallergic and autoimmune subtypes. Given that not all patients can be controlled with current treatment regimens, including anti-IgE monoclonal antibodies, a better understanding of the immune pathways involved in CSU may enable the repurposing of monoclonal antibodies used for other dermatologic diseases (e.g., Th2 and Th17 inhibitors). Therefore, we investigated the implicated immune cells and pathways by reanalyzing publicly available transcriptomic data.

**Methods:**

Microarray data of CSU and healthy control (HC) skin and blood were obtained from the Gene Expression Omnibus (GSE72542, GSE57178). Differentially expressed genes were defined as a false discovery rate <0.05 and a |log_2_ fold change| ≥1. Pathway analyses were conducted using ToppGene and KEGG. Cell-type enrichment was determined by CIBERSORT and xCell and was correlated with clinical characteristics.

**Results:**

Th2 (IL-4/13 signaling) and Th17-related (IL-17/23 signaling) pathways were upregulated in lesional compared to non-lesional and HC samples. In non-lesional versus lesional samples, CIBERSORT analysis revealed increased regulatory T-cells (Treg) and resting mast cells. xCell analysis established that Th1 and Th2 scores were not significantly different between lesional and HC samples. However, Th2 scores in both lesional and non-lesional samples correlated positively with disease severity. Few differentially expressed genes and pathways were identified between CSU and HC blood samples.

**Conclusion:**

Our results support the involvement of Th2 and Th17-related genes and pathways in CSU. Th2 scores associate with disease severity, which indicates the clinical relevance of these findings. Increased resting mast cell and Treg scores in non-lesional samples may suggest local suppression of wheal formation. Moreover, disease activity seemed to be restricted to the skin as there were limited findings from blood. Larger studies using next-generation sequencing will be helpful to confirm these results.

## Introduction

Chronic spontaneous urticaria (CSU) is defined by the presence of wheals and/or angioedema occurring in the absence of specific external stimuli and persisting for ≥ 6 weeks (1). CSU is associated with significant morbidity due to intractable pruritus that decreases quality of life by disrupting sleep and work/school productivity (2). Up to 55% of CSU cases are refractory to first- and second-line therapies consisting of second generation H1-antihistamines at standard and up to fourfold doses, respectively (1, 3, 4). In addition, up to 30% of patients requiring the third-line therapy, omalizumab, do not exhibit a complete response (5).

There has been a paradigm shift in the understanding of the pathogenesis of CSU and current evidence suggests that most CSU cases have an autoimmune etiology (6, 7). Autoimmune CSU involves either type I hypersensitivity (autoallergic type) attributable to autoallergic IgE antibodies or type IIb hypersensitivity (autoimmune type) attributed to anti-IgE or anti-IgE receptor (FcεRI) IgG autoantibodies (7, 8). Although the immunological events eliciting these antibodies are poorly understood, they result in mast cell/basophil degranulation and the release of vasoactive and inflammatory mediators and cytokines (e.g., histamine), which precipitate wheals and/or angioedema (9).

Several studies support the involvement of dysfunction of adaptive cellular immunity in the immune pathogenesis of CSU (10, 11). Current data on cell-types and cytokines involved in CSU is limited and largely obtained by immunohistochemistry, enzyme-linked immunosorbent assay, or flow cytometry (12–16). While it is plausible that specific subsets of Th cells may underlie autoallergic (IgE antibodies) versus autoimmune (IgG antibodies) subtype of CSU, it remains to be explored.

Although studies exist examining the immunopathogenesis of CSU, few studies use transcriptomics to broadly investigate the aberrant immune response in CSU. Transcriptomics enables the robust identification of novel biomarkers and the elucidation of disease pathogenesis and subsequent identification of actionable targets. A novel technique termed RNA deconvolution enables the *in silico* quantification of various cell-types from transcriptomic data of a heterogeneous cell population. CIBERSORT is one such RNA deconvolution technique that employs linear support vector regression to quantify relative proportions of cell types (17). Another *in silico* method of cell-type quantification is xCell which determines enrichment scores of up to 64 cell-types (18). Recently, CIBERSORT and xCell have been used to identify key differences in the microenvironment of various subtypes of basal cell carcinoma (19). A combination of xCell and CIBERSORT has also been used to characterize immune cell infiltration across various human tissues (20).

Driven by the hypothesis that Th2 immunity is important for B-cell phenotype switch and production of IgE, IgG1, and IgG4 antibodies, and that Th1/Th17 immunity is important for IgG2/3 commitment, we sought to investigate CSU immunopathogenesis using novel transcriptomic techniques [i.e., CIBERSORT (17) and xCell (18)] applied to publicly available microarray data to broadly characterize the immunological response and to correlate findings with patients’ clinical features.

## Materials and Methods

### Data Acquisition

Transcriptomic data acquired by microarray were obtained from the Gene Expression Omnibus (GEO) (21). From Giménez-Arnau et al. (GEO accession: GSE72542) (22), data was obtained from 9 CSU [lesional (wheal) and non-lesional (unaffected region of skin)] and 7 healthy control (HC) skin biopsies and 20 CSU and 10 HC blood samples. From Patel et al. (GEO accession: GSE57178) (23), data on 6 CSU (lesional and non-lesional) and 5 HC skin biopsies were analyzed. When available, associated clinical data including demographics, IgE levels, and autologous serum skin test (ASST) were extracted. Weekly urticaria activity scores (UAS7), a patient reported measure of disease severity evaluated by the number of wheals and severity of pruritus, were also extracted. Using this data, the following comparisons were designed: lesional versus HC skin, lesional versus non-lesional skin, and CSU versus HC blood.

### Pathway Analysis

Differentially expressed genes (DEGs) were determined using the GEO2R (21) online tool and were defined as genes with at least a twofold change in expression (|logFc| ≥1) and a false discovery rate (Q) less than 0.05. DEGs were determined separately for both datasets for each comparison, and gene lists were combined for pathway analyses. Pathway analyses were conducted using the ToppGene (24) and KEGG (25) online tools.

### Unsupervised Clustering and Violin Plots

DEG analysis was performed by The McGill Genome Centre, Montreal, Quebec. Datasets were analyzed separately. For each dataset, the normalized expression values were downloaded from the GEO database using the *getGEO* function from the *GEOquery* package (26), and linear models were fit to each probe or probeset using *Limma* (27). To integrate both datasets, the most differentially expressed probe or probeset per gene was retained, then only the common genes between both arrays were selected for a total of 15,940 genes. The package *MetaVolcanoR* (28) combined *p*-values from the separate datasets using Fisher’s method, and reported the logFc as the average logFc of both datasets. For visualization, expression values were transformed to z-scores independently for each dataset. With the z-scores, unsupervised hierarchical clustering was performed using the *Pheatmap* package (29) using the top 50 most upregulated genes (ranked by *Q*-value). Select DEGs were presented by violin plots.

### Cell-Type Estimation

RNA deconvolution was performed with CIBERSORT using the standard leukocyte gene signature matrix (LM22) and 100 permutations to estimate the relative proportions of 22 cell-types (17). Cell abundance scores were determined by xCell using the standard 64 cell-type signature (18). Absolute CIBERSORT and xCell scores were analyzed between comparisons by bootstrapping, a non-parametric resampling test, using 100,000 iterations. Differences of means were summed across the 100,000 comparisons to obtain a *p*-value. *P*-values were corrected by multiple hypothesis testing to give the Q via the Benjamini-Hochberg method. Correlations between cell-types and clinical characteristics were evaluated using Spearman’s rho and presented with a *p*-value.

### Statistical Analyses

*P*-values and *Q*-values less than 0.05 were considered significant. All statistical tests were performed on RStudio version 1.4.1717 and graphs and figures were rendered using the *Ggplot2* package (30).

## Results

### Lesional Versus Healthy Control Skin: Differentially Expressed Genes and Unsupervised Hierarchal Clustering

**FIGURE 1:**
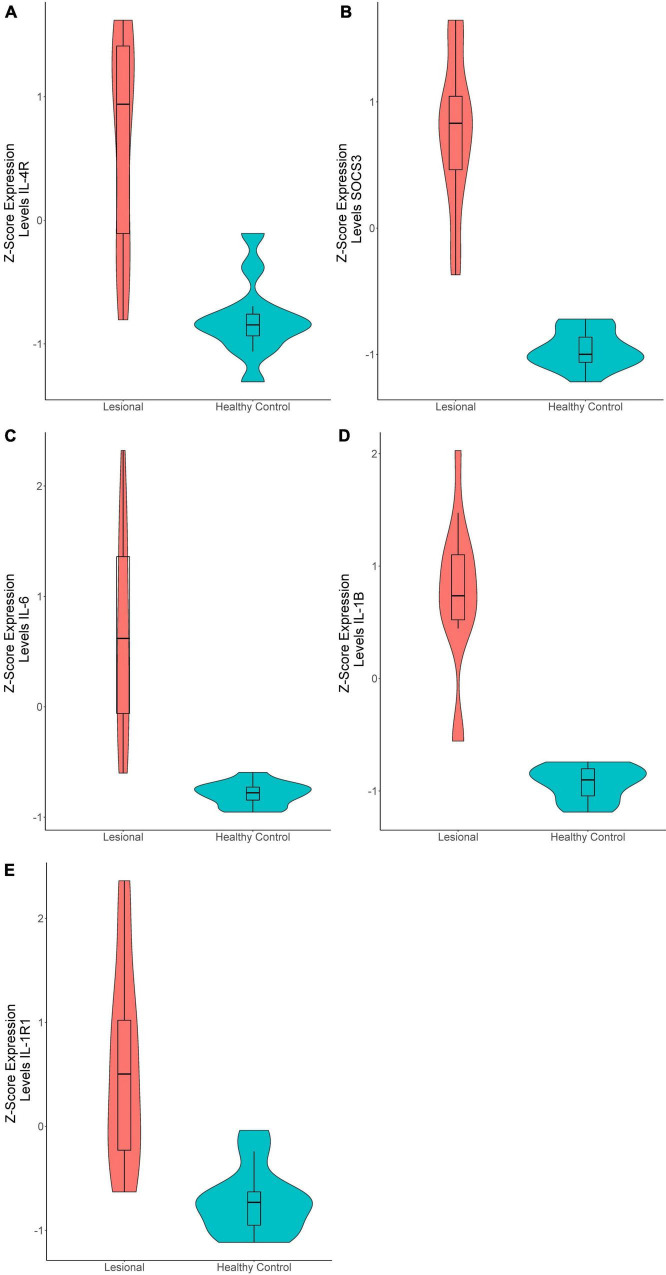
Violin plots of differentially expressed genes in lesional versus healthy control samples. **(A)** *IL-4R* (*Q* = 8.86E-7, logFc = 1.22), **(B)** *SOCS3* (*Q* = 7.06E-11, logFc = 2.27), **(C)** *IL-6* (*Q* = 3.12E-6, logFc = 2.51), **(D)** *IL-1B* (*Q* = 1.98E-8, logFc = 2.03), **(E)** *IL-1R1* (*Q* = 1.48E-6, logFc = 1.01).

**FIGURE 2:**
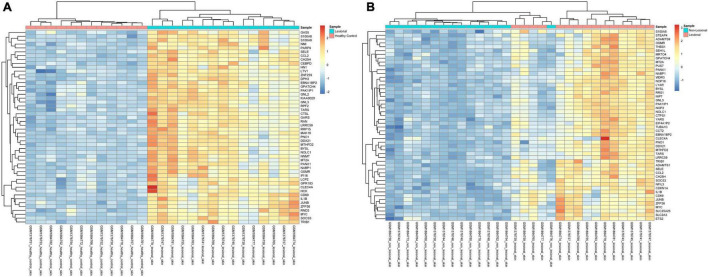
Unsupervised hierarchical clustering of the top 50 most upregulated genes in **(A)** lesional versus healthy control samples and **(B)** in lesional versus non-lesional samples. The color key indicates gene expression as a z-score.

In the 15 lesional versus 12 HC samples pooled from the two datasets, 270 genes were significantly upregulated and 81 were significantly downregulated (Supplementary Table 1). Interestingly, the Th2-related genes *IL-4R* (*Q* = 8.86E-7, logFc = 1.22, Figure 1A) and *SOCS3* (*Q* = 7.06E-11, logFc = 2.27, Figure 1B) were upregulated, as well as the Th17-related gene *IL-6* (*Q* = 3.12E-6, logFc = 2.51, Figure 1C). The proinflammatory gene *IL-1B* (*Q* = 1.98E-8, logFc = 2.03, Figure 1D) and its receptor *IL-1R1* (*Q* = 1.48E-6, logFc = 1.01, Figure 1E) were also upregulated. Unsupervised hierarchical clustering of the top 50 upregulated genes revealed two distinct groups between lesional CSU and HC samples with 100% group membership for each (Figure 2A).

### Lesional Versus Healthy Control Skin: Pathways

In Giménez-Arnau et al. (22) dataset 1,051 genes were significantly upregulated, and 863 genes were downregulated. In Patel et al. (23) dataset 246 genes were significantly upregulated and 20 were downregulated.

**FIGURE 3:**
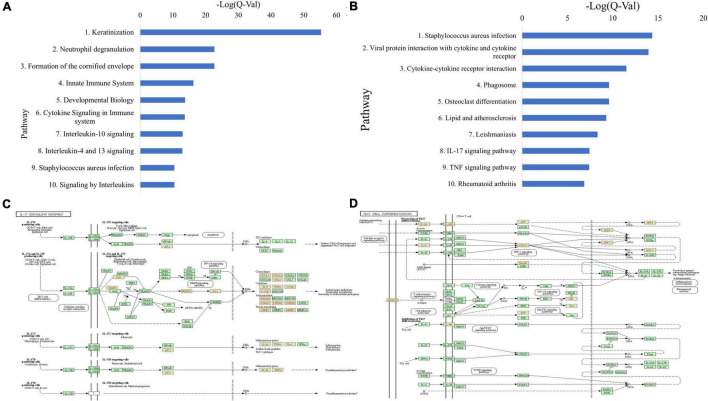
**(A)** Top 10 upregulated ToppGene pathways based on *Q*-value, in CSU lesional skin versus HC skin. **(B)** Top 10 upregulated KEGG pathways based on *Q*-value, in CSU lesional skin versus healthy control skin. **(C)** Upregulated genes (in red) overlaid on the KEGG IL-17 signaling pathway from lesional versus HC skin biopsies. **(D)** Upregulated genes (in red) overlaid on the KEGG Th17 cell differentiation pathway from lesional versus HC skin biopsies.

ToppGene pathway analysis (Figure 3A and Supplementary Table 2) of the combined upregulated gene lists revealed upregulated keratinization (*Q* = 4.62E-56), neutrophil degranulation (*Q* = 1.69E-23), innate immune system (*Q* = 4.50E-17), cytokine signaling in immune system (*Q* = 1.82E-14), staphylococcus aureus infection (*Q* = 2.85E-11), and signaling by interleukins (*Q* = 3.05E-11). Th2-related pathways including IL-4 and IL-13 mediated signaling (*Q* = 1.03E-13) and IL-18 signaling (*Q* = 2.05E-7) were upregulated. Additionally, IL-10 signaling (*Q* = 8.42E-14), an immunosuppressive pathway, and IL-12 mediated signaling (*Q* = 3.80E-3), a Th1 polarizing cytokine, were upregulated (Supplementary Table 2 and Supplementary Table 3). Other upregulated pathways include IL-23 mediated signaling events (*Q* = 1.04E-3), an activator directly upstream of the Th17 response, as well as IL-17 signaling (*Q* = 3.60E-6), and IL-6 mediated signaling events (*Q* = 8.28E-4) (Supplementary Table 3).

Upregulated KEGG pathways comprise staphylococcus aureus infection (*Q* = 4.42E-15), cytokine-receptor interaction (*Q* = 3.05E-12), and TNF signaling (*Q* = 4.02E-8) (Figure 3B and Supplementary Table 4). In line with the ToppGene pathway analysis, Th17-related pathways were upregulated including IL-17 signaling (*Q* = 3.75E-8) and Th17 cell differentiation (*Q* = 0.017) (Supplementary Table 3). Genes upregulated in the IL-17 signaling pathway were mostly downstream and clustered in the autoimmune pathology, neutrophil recruitment, and immunity to extracellular pathogens branches (Figure 3C), whereas those in the Th17 cell differentiation pathway were distributed throughout the pathway (Figure 3D). Individual analyses of both datasets separately were largely consistent with the presented results.

### Lesional Versus Non-lesional Skin: Differentially Expressed Genes and Unsupervised Hierarchal Clustering

**FIGURE 4:**
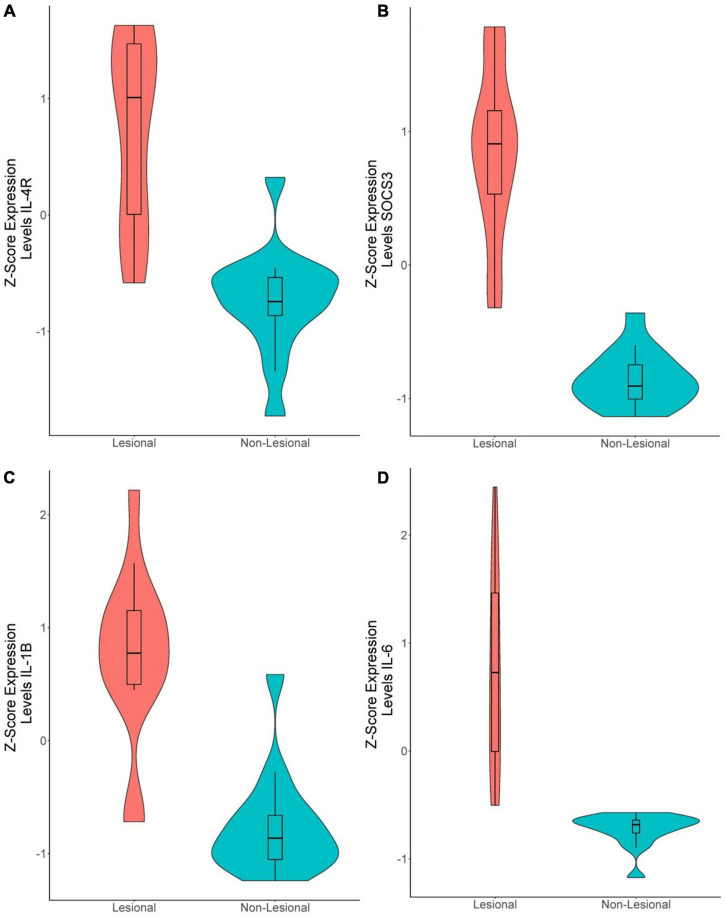
Violin plots of differentially expressed genes in lesional versus non-lesional samples. **(A)** *IL-4R* (*Q* = 1.49E-6, logFc = 1.08), **(B)** *SOCS3* (*Q* = 9.48E-11, logFc = 2.14), **(C)** *IL-1B* (*Q* = 4.89E-7, logFc = 1.60). **(D)** *IL-6* (*Q* = 1.86E-6, logFc = 2.52).

In the 15 lesional versus 15 non-lesional samples, 142 genes were significantly upregulated, and 20 were significantly downregulated (Supplementary Table 1). *IL-4R* was upregulated (*Q* = 1.49E-6, logFc = 1.08, Figure 4A), as well as *SOCS3* (*Q* = 9.48E-11, logFc = 2.14, Figure 4B), *IL-1B* (*Q* = 4.89E-7, logFc = 1.60, Figure 4C), and *IL-6* (Q = 1.86E-6, logFc = 2.52, Figure 4D). Unsupervised hierarchical clustering of the top 50 upregulated genes revealed three groups: one consisting of only non-lesional samples, one consisting of only lesional samples, and another of four lesional and one non-lesional samples (Figure 2B).

### Lesional Versus Non-lesional Skin: Pathways

In the Giménez-Arnau et al. (22) dataset 147 genes were significantly upregulated, and 89 genes were downregulated. In Patel et al. (23) dataset 241 genes were significantly upregulated and 19 were downregulated.

**FIGURE 5:**
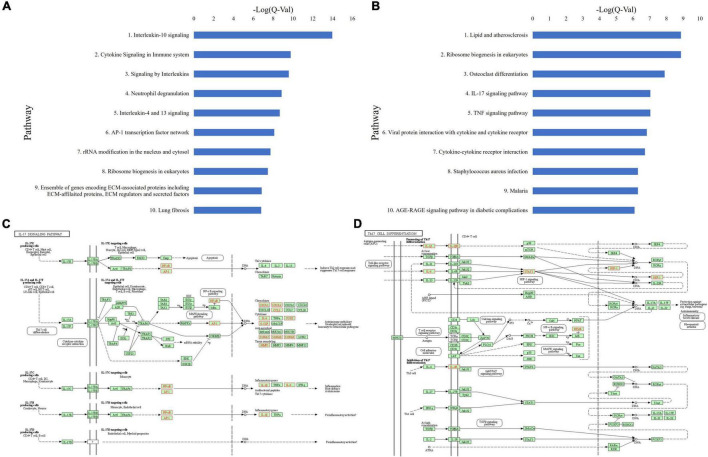
**(A)** Top 10 upregulated ToppGene pathways based on *Q*-value, in CSU lesional skin versus non-lesional skin. **(B)** Top 10 upregulated KEGG pathways based on *Q*-value, in CSU lesional skin versus non-lesional skin. **(C)** Upregulated genes (in red) overlaid on the KEGG IL-17 signaling pathway from lesional versus non-lesional skin biopsies. **(D)** Upregulated genes (in red) overlaid on the KEGG Th17 cell differentiation pathway from lesional versus non-lesional skin biopsies.

Similarly, ToppGene pathway analysis of the combined upregulated gene lists (Figure 5A and Supplementary Tables 5,6) revealed elevated IL-10 signaling (*Q* = 1.05E-14), cytokine signaling in immune system (*Q* = 1.70E-10), signaling by interleukins (*Q* = 2.60E-10), neutrophil degranulation (*Q* = 1.38E-9), IL-4 and IL-13 signaling (*Q* = 2.15E-9), IL-18 signaling (*Q* = 1.72E-4), IL-17 signaling (*Q* = 1.04E-3), IL-23 mediated signaling events (*Q* = 5.59E-4), IL-6 mediated signaling events (*Q* = 1.28E-3), and IL-12 mediated signaling events (*Q* = 2.57E-2). Additionally, the IL-4 mediated signaling events pathway (*Q* = 2.89E-2) was also upregulated (Supplementary Table 6).

KEGG pathway analysis of lesional versus non-lesional skin identified upregulated cytokine-receptor interaction (*Q* = 1.92E-7), TNF signaling (*Q* = 9.20E-8), and staphylococcus aureus infection (*Q* = 5.06E-7) (Figure 5B and Supplementary Table 7). Moreover, IL-17 signaling (*Q* = 9.20E-8) and Th17 cell differentiation (*Q* = 0.019) were upregulated (Supplementary Table 6). Upregulated genes in the IL-17 signaling (Figure 5C) and Th17 differentiation (Figure 5D) pathways were distributed similar to the lesional versus HC samples, albeit with less coverage of the pathway. Analysis of both individual datasets were mostly consistent with the union analysis.

### Chronic Spontaneous Urticaria Versus Healthy Control Blood: Pathways and Differentially Expressed Genes

Five genes were upregulated and seven downregulated in the 20 CSU versus 10 HC blood samples. ToppGene pathway analysis of upregulated genes revealed enriched Th1 and Th2 cell differentiation (*Q* = 0.033), Th17 cell differentiation (*Q* = 0.027), IL-5 signaling pathway and Th1/Th2 differentiation (*Q* = 0.027). Similarly, KEGG analysis demonstrated enriched Th1 and Th2 cell differentiation (*Q* = 0.045) and Th17 cell differentiation (*Q* = 0.045). These results must be interpreted with caution, however, because *HLA-DRB3* (logFc = 1.08, *Q* = 0.025) was the only upregulated gene involved in each of these pathways.

### Lesional Versus Healthy Control Skin: Cell-Type Enrichment

**FIGURE 6:**
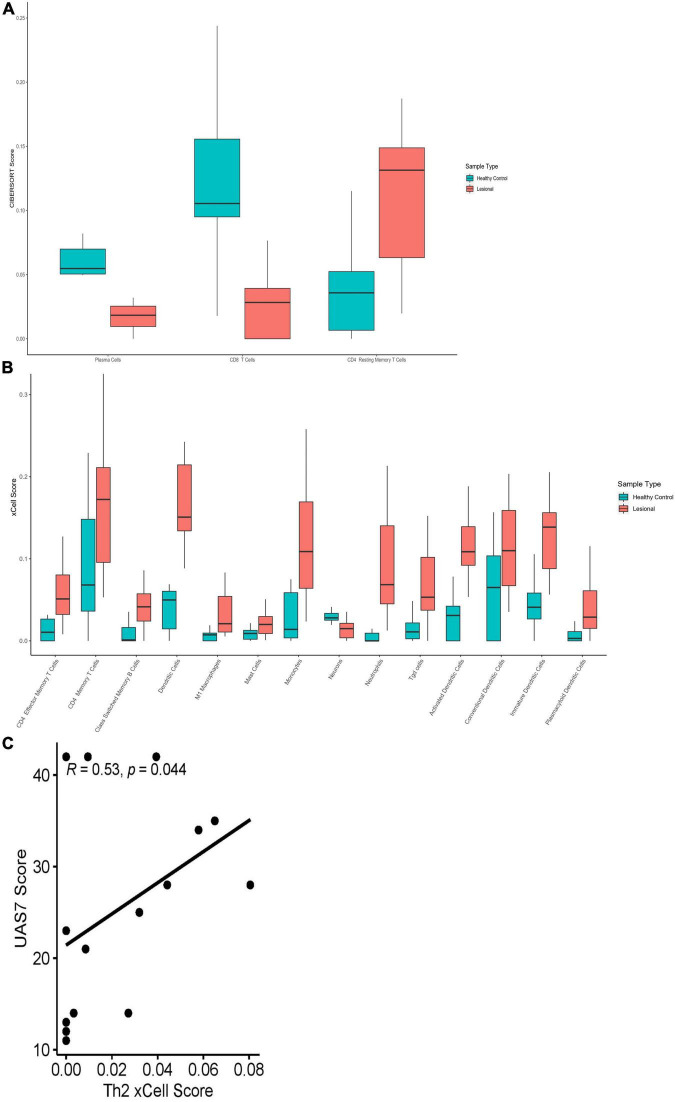
**(A)** CIBERSORT scores of plasma cells (*Q* = 0.029), CD8+ T-Cell (*Q* = 0.0066) and CD4+ resting memory T-Cell (*Q* = 0.0081) in lesional versus healthy control skin samples. **(B)** xCell scores for CD4+ effector memory T-Cells (*Q* = 0.020), CD4+ memory T-cells (*Q* = 0.039), class-switched memory B-cells (*Q* = 0.015), Tgd cells (*Q* = 0.015), M1 macrophages (*Q* = 0.022), mast cells (*Q* = 0.039), monocytes (*Q* = 0.015), neutrophils (*Q* = 0.0055), dendritic cells (*Q* = 0.0010), activated dendritic cells (*Q* = 0.0010), conventional dendritic cells (*Q* = 0.047), immature dendritic cells (*Q* = 0.0050), plasmacytoid dendritic cells (*Q* = 0.037) and neurons (*Q* = 0.013) in lesional versus healthy control skin samples. **(C)** Th2 xCell abundance scores correlated with UAS7 scores in lesional skin samples, presented with Spearman’s rho coefficient.

RNA deconvolution of 15 lesional and 12 HC skin samples was performed by CIBERSORT (Figure 6A). In lesional compared to HC samples, plasma cells (*Q* = 0.029) and CD8+ T-cells (*Q* = 0.0066) were decreased, whereas CD4+ resting memory T-cells (*Q* = 0.0081) were increased. Eosinophils were only detected in some lesional skin samples, but not statistically significantly greater than in HC samples. No significant differences were observed in B-cells, natural killer (NK) cells, macrophages, DCs, neutrophils, or Tregs.

Cell abundance scores determined by xCell were compared between lesional and HC skin samples (Figure 6B). CD4+ effector memory T-cells (Tem) (*Q* = 0.020), CD4+ memory T-cells (*Q* = 0.039), common lymphoid progenitor cells (*Q* = 0.025), and class switched memory B-cells (*Q* = 0.015) had increased cell abundance scores in lesional compared to HC samples. No significant differences were observed in Th1 (*Q* = 0.30) or Th2 (*Q* = 0.55) cells; however, gamma delta T-cells (Tgd) had elevated cell abundance scores in lesional samples (*Q* = 0.015). Th2 scores in lesional samples correlated moderately with UAS7 (Figure 6C, Spearman’s rho = 0.53, *p* = 0.044). Innate immune cells including M1 macrophages (*Q* = 0.022), mast cells (*Q* = 0.039), monocytes (*Q* = 0.015), and neutrophils (*Q* = 0.0055) had increased scores in lesional samples in contrast to HC samples. Similarly, total dendritic cells (DC) were elevated in lesional versus HC samples (*Q* = 0.0010), including activated dendritic cells (aDC) (*Q* = 0.0010), conventional dendritic cells (cDC) (*Q* = 0.047), immature dendritic cells (iDC) (*Q* = 0.0050), and plasmacytoid dendritic cells (pDC) (*Q* = 0.037). Interestingly, neurons (*Q* = 0.013) were decreased in lesional compared to HC samples. While basophils were detected in both lesional and HC samples, abundance scores were not significantly different.

### Lesional Versus Non-lesional Skin: Cell-Type Enrichment

**FIGURE 7:**
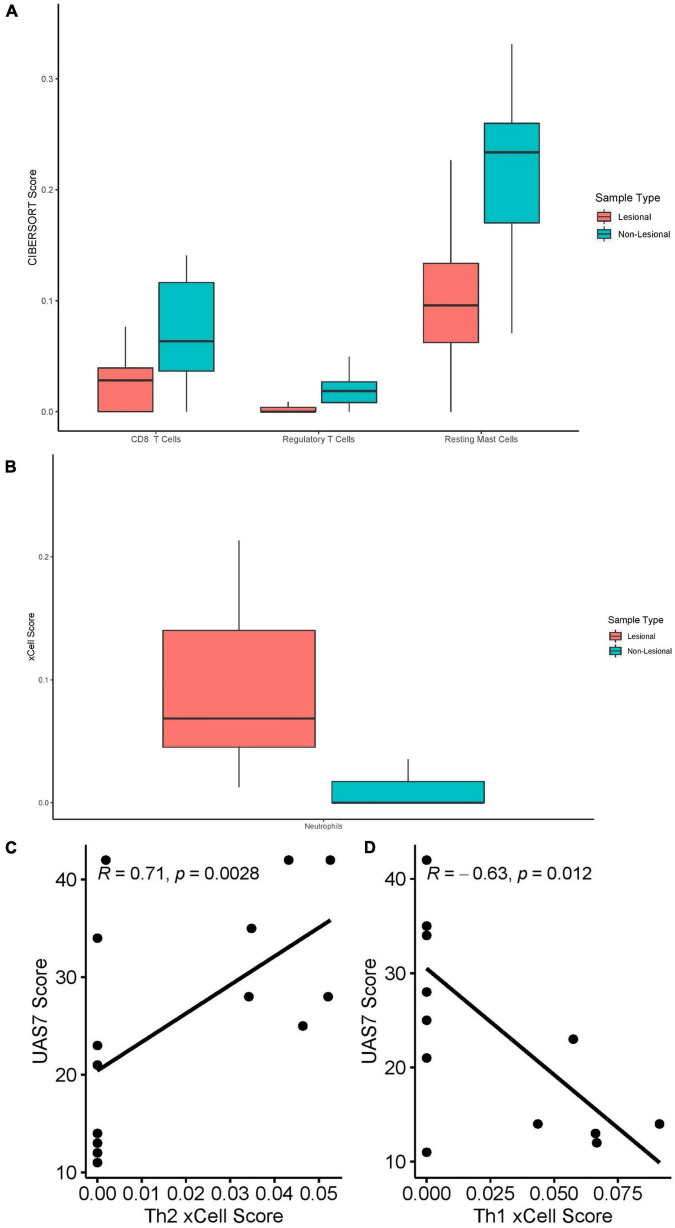
**(A)** CIBERSORT scores of CD8+ T-cells (*Q* = 0.039), Tregs (*Q* = 0.014) and resting mast cells (*Q* = 0.014) in lesional versus non-lesional skin samples. **(B)** Neutrophil xCell score (*Q* = 0.012) in lesional versus non-lesional skin samples. **(C)** Th2 xCell scores in non-lesional skin samples correlated with UAS7 scores, presented with Spearman’s rho coefficient. **(D)** Th1 cell xCell score in non-lesional skin samples, presented with Spearman’s rho coefficient.

CIBERSORT RNA deconvolution of 15 lesional versus 15 matched non-lesional samples (Figure 7A) revealed elevated CD8+ T-cells (*Q* = 0.039) in non-lesional samples. Interestingly, Tregs (*Q* = 0.014) and resting mast cells (*Q* = 0.014) were increased in non-lesional compared to lesional samples. Eosinophils were only detected in some lesional skin samples, but not statistically significantly greater than non-lesional samples. We did not detect any significant differences in B-cells, NK cells, macrophages, DCs, or neutrophils between lesional and non-lesional samples on CIBERSORT.

xCell analysis of lesional versus non-lesional skin samples (Figure 7B) revealed only elevated neutrophil cell abundance scores (*Q* = 0.012). Moreover, Th2 cell abundance scores of non-lesional samples correlated strongly with UAS7 scores (Figure 7C, Spearman’s rho = 0.71, *p* = 0.0028), whereas Th1 cell abundance scores correlated negatively with UAS7 scores (Figure 4D, Spearman’s rho = –0.63, *p* = 0.012). Basophils were detected in both groups but were not significantly different.

### Chronic Spontaneous Urticaria Versus Healthy Control Blood: Cell-Type Enrichment

**FIGURE 8:**
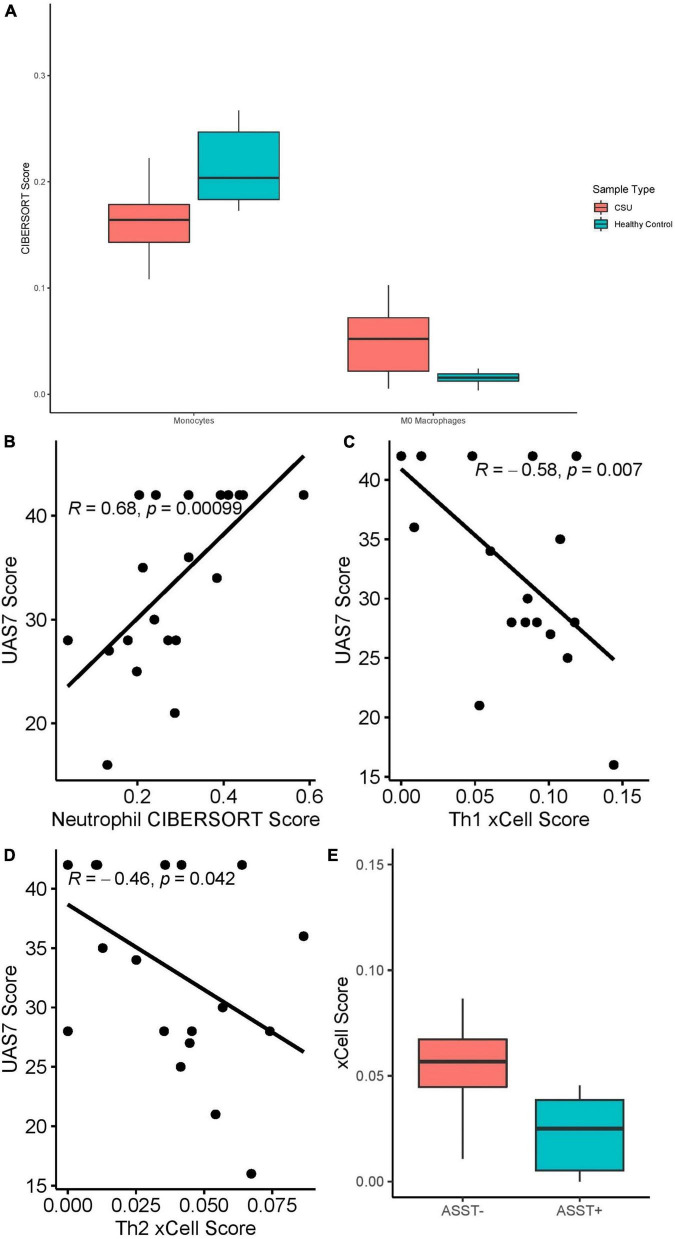
**(A)** Monocyte (*Q* = 0.018) and M0 macrophage (*Q* = 0.018) CIBERSORT scores in CSU versus healthy control blood samples. **(B)** Neutrophil CIBERSORT scores in CSU blood samples correlated with UAS7 scores, presented with Spearman’s rho coefficient. **(C)** Th1 xCell scores in CSU blood samples correlated with UAS7 scores, presented with Spearman’s rho coefficient. **(D)** Th2 xCell score in CSU blood samples correlated with UAS7 score, presented with Spearman’s rho coefficient. **(E)** Th2 xCell score in ASST+ versus ASST– CSU blood samples (*p* = 0.019).

RNA deconvolution using CIBERSORT of 20 CSU blood samples and 10 HC blood samples (Figure 8A) revealed elevated macrophages (*Q* = 0.018) and decreased monocytes (*Q* = 0.018) in CSU blood compared to HC blood. No eosinophils were detected in either CSU or HC blood and no significant differences were detected in other cell-types. Although neutrophils were not enriched in CSU blood samples compared to HC blood, neutrophil CIBERSORT scores strongly positively correlated with UAS7 scores (Figure 8B, Spearman’s rho = 0.68, *p* = 0.00099).

xCell analysis did not detect differences in cell-type abundance scores between CSU and HC blood samples. Among CSU blood samples, Th1 (Figure 8C, Spearman’s rho = –0.58, *p* = 0.007) and Th2 (Figure 8D, Spearman’s rho = –0.46, *p* = 0.042) cell abundance scores correlated negatively with UAS7 scores. Basophils were only detected in CSU samples but not statistically significantly greater than in HC samples. Moreover, Th2 cells were significantly decreased in ASST+ samples compared to ASST– samples (Figure 8E, *p* = 0.019), but no difference was observed in Th1 cells (*p* = 0.82).

### Individual Dataset Analyses: Cell-Type Enrichment

The Giménez-Arnau et al. (22) and Patel et al. (23) skin datasets were analyzed individually with CIBERSORT and xCell (Supplementary Tables 8, 9, respectively). Additionally, CIBERSORT and xCell results were compared between the two datasets (Supplementary Table 10). No significant differences in any of the 64 cell-types measured by xCell were detected between lesional samples from the two datasets. Only naïve B-cells (*Q* = 0.014) and resting DCs (*Q* = 0.014) differed on CIBERSORT analysis. However, significant differences were identified in five cell-types by CIBERSORT and 16 cell-types by xCell in HC samples and six cell-types by CIBERSORT and 13 cell-types by xCell in non-lesional samples.

## Discussion

Recently, the role of Th2 dysregulation has been proposed as a potential driver of CSU pathogenesis in at least a subset of CSU patients (11, 31). Th2 cells and downstream cytokines IL-4/5/6/10/13 are important for B cell differentiation and IgE production as well as eosinophil, mast cell, and basophil recruitment/activation, which are the common laboratory and histologic hallmarks of CSU (31). This knowledge and a successful proof of concept case series of 6 refractory CSU patients responding to dupilumab (32), an IL4-Rα inhibitor, stimulated the initiation of phase III clinical trial of dupilumab in CSU.

Th2 response is not only important for IgE antibody production, but also stimulates IgG1 complement binding and IgG4 antibodies underlying other autoimmune conditions (31). Interestingly, one study of 22 CSU patients reported that anti-FcεRI-α antibodies were predominantly of IgG1 and IgG3 subtype, which suggests that Th2 and possibly Th17-mediated IgG class switch may be of importance (8).

We showed that the IL-4, IL-13, and IL-18 cytokine signaling pathways were upregulated in lesional CSU biopsies versus both HC and non-lesional samples, which suggest a role in the wheal response. Other studies have shown that cytokines that initiate the Type 2 response (IL-25, IL-33, and thymic stromal lymphopoietin) (33) and downstream Th2 cytokines (IL-4, IL-5, and IL-13) (12, 34) are elevated in CSU wheal biopsies compared to control skin biopsies. Although reports of increased IL-18 in serum of CSU patients are inconsistent (35, 36), we identified involvement of the IL-18 signaling pathway in CSU skin. IL-18 is a pleiotropic cytokine whose effects depend on the cytokine environment, which in the presence of IL-2 and/or IL-3 can stimulate production of Th2 cytokines (IL-4 and IL-13) and histamine (37). Further, we found increased IL-10 signaling pathway in lesional CSU samples compared to non-lesional and HC skin samples. IL-10 is an immunosuppressive cytokine predominantly secreted by Th2 and Tregs (38) and mixed reports exist on serum IL-10 levels in CSU patients versus controls (39, 40). However, *IL-10* expression was shown to be increased in CSU lesions and a CSU mouse model overexpressing *IL-10* exhibited increased pruritus and increased *IL-2*, *IL-4*, and *IFN*-γ expression, driven by JAK/STAT signaling (41). It is unclear whether the upregulation of the *IL-10* signaling pathway we observed is contributing to CSU inflammation by driving Th2 cytokines (e.g., by IL-4) or whether it is an insufficient immunosuppressive response.

Contrary to a previous study reporting increased Th2 cells in CSU lesions (42), we did not detect a significant increase in Th2 cell abundance scores in lesional versus non-lesional and HC samples. However, Th2 cell abundance scores were significantly positively correlated with UAS7 scores in both lesional and non-lesional CSU samples, whereas they were negatively correlated with UAS7 scores in blood. This inverse relationship may be indicative of Th2 cell tissue extravasation and recruitment to skin associated to worsening disease activity. Th2 cell abundance scores were increased in ASST negative patients suggesting that it may be more important in autoallergic as opposed to autoimmune CSU cases. However, further larger studies analyzing IgE levels and gold standard methods of confirming autoimmune CSU are needed (43).

The Th17 response has been implicated in the pathogenesis of several autoimmune diseases, including CSU. Notably, Th17 cells promote B-cell proliferation and IgG class-switching (44). In this study we identified several upregulated Th17-related pathways including IL-17 signaling, Th17 cell differentiation, IL-6 and IL-23 mediated signaling events in lesional versus both non-lesional and HC samples. Key Th17 genes (e.g., *IL-6*) were also upregulated in lesional samples compared to non-lesional and HC samples. IL-17 and IL-23 have been shown to be elevated in the serum of CSU patients compared to controls and positively correlate with UAS7 scores (45). A recent open label trial documented significant symptom improvement of refractory CSU by secukinumab, an IL-17α inhibitor (46), which underscores the therapeutic relevance of the Th17 pathway in CSU. Of note, Th17 cells are important for IgG2 and IgG3 phenotype switch (44). Because xCell and CIBERSORT do not measure Th17 cells, we were unable to determine if Th17 cells were enriched in CSU samples and if they correlated with clinical characteristics.

We found little evidence for the involvement of Th1 cells in the CSU samples studied. Th1 cells were not enriched in any samples and few Th1 pathways were upregulated (e.g., IL-12 mediated signaling). A Th1 response is usually associated with ASST positivity (39), but in our study Th1 cell abundance scores were not different between ASST+ and ASST– patients. However, Th1 cell abundance scores correlated negatively with UAS7 scores in non-lesional and blood samples.

Previous reports document decreased Tregs in CSU blood versus HCs (16, 47). *In vivo* and *in vitro* evidence indicate a role for Tregs in the suppression of mast cell degranulation via OX40-OX40L signaling (48). While we did not observe Treg suppression in CSU blood samples, we observed Tregs and resting mast cells upregulation in non-lesional skin compared to lesional, which suggests a role for Tregs in the inhibition wheals by suppressing mast cells into a resting state. Interestingly, in the presence of IL-6, scarce Th1/Th2 cytokines, and activated mast cells, Treg suppression can be circumvented, inducing a Th17 response (49).

In tandem with altered adaptive immunity, dysregulation of innate immune cells contributes to the pathogenesis of CSU (15, 50). We found elevated *IL-1B* and its receptor *IL-1R1*, which are a pro-inflammatory cytokines involved in innate immunity (51), as well as several differences in innate immune cells. Mast cells are the predominant mediator of the wheal and flare response via histamine release and represent one of the effector cells of Th2 response; therefore, it is not surprising that resting (as opposed to active) mast cells were elevated in non-lesional compared to lesional skin. Neutrophil infiltration has been observed in the majority (94%) of CSU lesional biopsies (52) and blood neutrophil counts correlate negatively with CSU prognosis in children (53). Correspondingly, we found that neutrophil abundance scores in blood correlated positively with UAS7 and that the neutrophil degranulation pathway was upregulated in lesional samples compared to both HC and non-lesional skin. Our results reveal that all types of DCs detected by xCell (DCs, pDCs, iDCs, cDCs, and aDCs) were elevated in lesional compared HC samples. pDCs express FcεRIα and bind IgE. In CSU patients pDCs have impaired IFN-α secretion in response to TLR-9 stimulation (54), which is not restored by omalizumab treatment (55). Data on the involvement of other types of DCs in CSU are limited. Interestingly, in atopic dermatitis and psoriasis, cutaneous antigen presenting cDCs can polarize the immune response toward Th2 or Th1 cytokines, respectively (56).

Our study has some limitations. The low sample size of our study diminishes the power of our results and may be the reason why Th2 upregulation was not seen in lesional skin. It is plausible that larger studies using RNA sequencing will confirm Th2 and Th17 implication in CSU pathogenesis and may explain the phenotypic dichotomy seen clinically with Th2 signaling correlating with autoallergic subtype whereas Th17 pathway correlating with at least a subset of autoimmune CSU patients. Despite the low sample size of our study, other studies with similar sample sizes have identified important genes from a pooled re-analysis approach (57). Although several significant differences were observed between non-lesional and HC samples, lesional samples were largely consistent between the two datasets. Differences between HC cell composition may reflect differences in patient demographics, whereas differences in non-lesional samples may reflect differences in biopsy location or timing. Moreover, transcriptomic data of blood was only available from one dataset. The results of this study may not be generalizable to all CSU patients because both datasets included only patients who had failed treatment with antihistamines at standard doses. Because this work was done *in silico*, we are unable to validate DEGs by reverse transcription-polymerase chain reaction. Our data need to be interpreted with caution because deconvolution of similar cell-types is less precise and spillover of one cell-type into another may exist (58). Finally, we are limited by the quality of available transcriptomic data on CSU as all transcriptomic data analyzed were obtained by microarray, which is inferior to RNA sequencing at detecting and quantifying DEGs (59). Therefore, we recommend future studies using high throughput RNA sequencing to validate available microarray data.

## Conclusion

Taken together, we have identified several key pathways and immune cell infiltrates involved in CSU that contribute to the understanding of the pathogenesis of CSU. We identified a role for Th2 and Th17 dysregulation in CSU lesions, which may be actionable targets using available monoclonal antibodies. Our study found several cell-types elevated in CSU lesions compared to HCs and non-lesional samples, which can inform future studies on the roles of these cells in CSU pathogenesis. Finally, we established several correlations between cell infiltrates and disease activity, which suggest clinical relevance of our findings.

## Data Availability Statement

The datasets presented in this study can be found in online repositories. The names of the repository/repositories and accession number(s) can be found in the article/Supplementary Material.

## Author Contributions

PL, EN, IL, and CP designed the study. CP, PL, SG, and ML performed the data analysis. CP and SG prepared the figures and tables. EN, PL, IL, MB-S, and AG-A supervised the study. All authors contributed to writing and editing the manuscript.

## Conflict of Interest

EN was a consultant and speaker for Novartis and has received an investigator-initiated research grant from Novartis. MB-S was a consultant for Novartis. AG-A was a medical advisor for Uriach Pharma, Genentech, Novartis, FAES, GSK, Sanofi–Regeneron, Amgen, Thermo Fisher Scientific, Almirall, she has research grants supported by Uriach Pharma, Novartis, Grants from Instituto Carlos III- FEDER and performs educational activities for Uriach Pharma, Novartis, Genentech, Menarini, LEO-PHARMA, GSK, MSD, Almirall, Sanofi—Regeneron, AVENE. The remaining authors declare that the research was conducted in the absence of any commercial or financial relationships that could be construed as a potential conflict of interest.

## Publisher’s Note

All claims expressed in this article are solely those of the authors and do not necessarily represent those of their affiliated organizations, or those of the publisher, the editors and the reviewers. Any product that may be evaluated in this article, or claim that may be made by its manufacturer, is not guaranteed or endorsed by the publisher.

## Abbreviations

aDC: Activated Dendritic Cell
ASST: Autologous Serum Skin Test
cDC: Conventional Dendritic Cell
CSU: Chronic Spontaneous Urticaria
DC: Dendritic Cell
HC: Healthy Control
iDC: Immature Dendritic Cell
logFC: Log_2_ Fold Change
NK: Natural Killer Cell
pDC: Plasmacytoid Dendritic Cell
Q: False Discovery Rate
Tem: CD4+ Effector Memory T-Cell
Tgd: Gamma Delta T-Cell
Th: T-helper
Treg: Regulatory T-Cell
UAS7: Urticaria Activity Score Over 7 Days.
