# Supplementary material for: In silico Identification of Immune Cell-Types and Pathways Involved in Chronic Spontaneous Urticaria
Source: Front Med (Lausanne). 2022 Jul 7;9:926753. doi: 10.3389/fmed.2022.926753 (PMC9302568; doi:10.3389/fmed.2022.926753)
Supplement: Supplementary file 1 [file Data_Sheet_1.pdf]

Supplemental Table 1. Differentially expressed genes in lesional versus healthy control and lesional versus non-lesional skin, ranked by Q-value, combined data from both datasets.

Lesional Versus Healthy Control						Lesional Versus Non-Lesional					
Upregulated			Downregulated			Upregulated			Downregulated		
Gene Symbol	Log2 Fold Change	Q-Value	Gene Symbol	Log2 Fold Change	Q-Value	Gene Symbol	Log2 Fold Change	Q-Value	Gene Symbol	Log2 Fold Change	Q-Value
CCL2	3.46	4.16E-13	AHSA2	-1.13	2.97E-09	CCL2	3.31	1.06E-12	STARD9	-1.10	1.19E-09
CH25H	2.31	3.39E-11	EBF3	-1.00	3.75E-09	SELE	2.96	2.24E-11	OSR2	-1.26	1.95E-06
CD69	2.30	7.06E-11	BOC	-1.07	1.81E-08	CH25H	2.25	2.24E-11	ABCA6	-1.49	4.60E-06
SOCS3	2.27	7.06E-11	PIK3IP1	-1.10	2.49E-08	SOCS3	2.14	9.48E-11	CD34	-1.18	1.13E-05
SELE	2.91	1.67E-10	TNS1	-1.18	4.07E-08	BYSL	1.09	1.19E-09	ABCA9	-1.03	1.62E-05
CEBPD	1.02	1.39E-09	NUAK1	-1.26	4.40E-08	CD69	1.81	1.29E-09	ABCA8	-1.04	5.19E-05
NNMT	2.42	2.97E-09	VIPR1	-1.03	5.19E-08	DDX21	1.79	2.62E-09	SEPP1	-1.08	2.84E-04
DDX21	1.78	2.97E-09	RNPC3	-1.15	5.19E-08	NOLC1	1.32	3.05E-09	RERGL	-1.14	3.38E-04
BYSL	1.09	2.97E-09	HOXA6	-1.57	5.42E-08	OSMR	1.58	2.46E-08	LGR5	-1.43	8.69E-04
ZNF259	1.08	2.97E-09	CLDN5	-1.30	1.01E-07	RRS1	1.37	2.46E-08	KRT15	-1.14	1.04E-03
DPH3	1.05	3.39E-09	SUN1	-1.03	1.96E-07	PAK1IP1	1.24	3.07E-08	TIMP3	-1.05	1.08E-03
MT2A	2.30	4.48E-09	ANKRD36C	-1.47	2.44E-07	MT2A	2.01	3.10E-08	DCLK1	-1.19	1.22E-03

S100A8	4.28	4.61E-09	LDB2	-1.15	2.84E-07	GNL3	1.02	3.10E-08	ADH1C	-1.11	1.25E-03
CLEC4A	1.72	4.64E-09	RERG	-1.13	3.82E-07	CTPS1	1.24	3.52E-08	ANGPTL1	-1.37	1.85E-03
NMI	1.13	4.64E-09	HOXA5	-1.13	4.53E-07	WDR3	1.18	3.73E-08	SLC14A1	-1.32	3.37E-03
S100A9	3.25	6.42E-09	CD86	-2.23	4.77E-07	PNO1	1.20	4.48E-08	ADH1B	-1.14	3.71E-03
MAK16	1.07	6.42E-09	NKAPP1	-1.26	6.31E-07	NIP7	1.13	4.51E-08	ACTA2	-1.02	8.88E-03
JUNB	1.67	7.90E-09	HECTD4	-1.08	6.38E-07	MYC	1.63	4.51E-08	AQP5	-1.00	2.36E-02
OSMR	1.74	8.24E-09	OSR2	-1.46	6.73E-07	NOP2	1.25	4.95E-08	SLC12A2	-1.07	3.38E-02
LCP2	1.20	8.24E-09	MAP3K4	-1.12	7.94E-07	JUNB	1.40	4.95E-08	SCGB2A2	-1.22	4.83E-02
RAN	1.03	8.41E-09	TET1	-1.05	9.95E-07	PUS7	1.21	6.24E-08			
CTSL	2.71	1.01E-08	ZNF273	-1.30	1.54E-06	CLEC4A	1.42	6.24E-08			
MTHFD2	1.50	1.01E-08	ATP1B2	-1.07	1.86E-06	YARS	1.14	6.24E-08			
HN1	1.10	1.01E-08	DDX17	-1.18	3.03E-06	GPATCH4	1.06	6.24E-08			
GARS	1.10	1.01E-08	SOSTDC1	-1.20	3.22E-06	MTHFD2	1.38	7.75E-08			
PNO1	1.33	1.16E-08	TG	-1.27	3.27E-06	CDKN1A	1.07	9.34E-08			
GPATCH4	1.23	1.18E-08	TNNI2	-1.08	4.00E-06	SEH1L	1.06	9.70E-08			

KIAA0020	1.04	1.21E-08	ARHGEF28	-1.22	4.16E-06	TARS	1.06	1.13E-07			
GNL3	1.05	1.25E-08	SNORA37	-1.25	4.17E-06	CCT2	1.01	1.13E-07			
ZFP36	2.07	1.38E-08	RCAN2	-1.03	4.99E-06	NFIL3	1.13	1.29E-07			
PAK1IP1	1.23	1.49E-08	HBP1	-1.04	5.42E-06	SLC2A3	1.51	1.40E-07			
GAS5	1.05	1.49E-08	KRT15	-1.68	6.15E-06	TRIB1	1.18	1.40E-07			
LRRC59	1.52	1.53E-08	FAT3	-1.04	6.31E-06	ZFP36	1.76	1.44E-07			
PARP9	1.20	1.53E-08	VWF	-1.21	6.46E-06	ETS2	1.28	1.44E-07			
PANX1	1.55	1.62E-08	LMOD1	-1.26	6.79E-06	EIF4A1P2	1.01	1.87E-07			
IL1B	2.03	1.98E-08	CRHR1-IT1	-1.47	7.75E-06	MRTO4	1.09	2.06E-07			
NABP1	1.89	3.06E-08	TFCP2L1	-1.23	9.06E-06	ADAMTS1	1.55	2.21E-07			
HCK	1.35	3.06E-08	SOX6	-1.01	9.93E-06	NOP16	1.03	2.61E-07			
RRP15	1.10	3.06E-08	HIST1H1E	-1.13	1.47E-05	STEAP4	2.15	3.08E-07			
LTV1	1.07	3.67E-08	TBX18	-1.09	2.07E-05	THBS1	2.47	3.37E-07			
GPR183	1.86	3.68E-08	FOXS1	-1.07	2.07E-05	ADAMTS9	1.77	3.42E-07			
EBNA1BP2	1.22	3.90E-08	PPP6R1	-2.10	2.20E-05	S100A8	3.34	3.44E-07			

MYC	1.60	4.06E-08	KANK2	-1.02	2.36E-05	EBNA1BP2	1.09	3.55E-07			
TRIB1	1.31	4.06E-08	SYNPO2	-1.30	2.51E-05	TUBA1C	1.18	3.55E-07			
IFI16	1.35	4.08E-08	MYLK	-1.17	3.28E-05	LYAR	1.07	4.02E-07			
RPF2	1.03	4.61E-08	CKM	-1.09	3.86E-05	SLC25A25	1.18	4.33E-07			
NOLC1	1.18	4.70E-08	PGM5	-1.04	4.89E-05	PANX1	1.30	4.37E-07			
RND3	1.13	4.86E-08	SPEN	-1.91	4.94E-05	IL1B	1.60	4.89E-07			
TARS	1.09	4.86E-08	PDE5A	-1.03	4.97E-05	LRRC59	1.25	5.33E-07			
GNL2	1.04	4.91E-08	FZD4	-1.04	5.18E-05	NABP1	1.70	5.33E-07			
UTP14A	1.04	5.42E-08	RPA4	-1.88	5.64E-05	S100A9	2.37	6.28E-07			
LYAR	1.21	5.67E-08	VPS18	-2.03	1.03E-04	NNMT	1.62	6.47E-07			
SERPINB1	1.31	7.21E-08	AOC3	-1.26	2.22E-04	RIOK1	1.03	6.63E-07			
PUS7	1.20	7.39E-08	RERGL	-1.19	2.33E-04	SERPINB1	1.13	7.71E-07			
URB2	1.02	7.81E-08	TIMP3	-1.18	3.13E-04	TMEM2	1.14	7.72E-07			
THBS1	2.80	8.89E-08	LRRC2	-2.27	3.50E-04	DNAJA1	1.05	8.49E-07			
UAP1	2.44	9.67E-08	ACTA2	-1.34	3.75E-04	CYR61	1.74	9.38E-07			

TNFAIP6	3.78	1.10E-07	TPM2	-1.07	4.42E-04	PNP	1.62	9.83E-07			
CCR1	1.60	1.33E-07	KCNK5	-1.12	5.34E-04	CXCL1	1.32	9.88E-07			
WDR3	1.08	1.40E-07	MYOC	-1.20	7.98E-04	CTSL	2.02	1.01E-06			
SLC39A14	2.41	1.42E-07	ACTBL2	-1.10	8.61E-04	MPZL2	1.05	1.07E-06			
DNAJA1	1.21	1.42E-07	DES	-1.50	1.20E-03	SLC39A14	1.54	1.31E-06			
CYTIP	1.42	1.73E-07	ATP1B1	-1.11	1.34E-03	IL4R	1.08	1.49E-06			
DDX10	1.11	1.73E-07	MYL9	-1.12	1.43E-03	HSPH1	1.30	1.54E-06			
HSPA4	1.03	1.97E-07	EPB42	-1.05	1.49E-03	GPR183	1.41	1.79E-06			
HSP90AA1	1.01	2.03E-07	RHPN2	-1.13	1.86E-03	IL6	2.52	1.86E-06			
YARS	1.07	2.10E-07	FRZB	-1.13	2.11E-03	IFI16	1.02	1.95E-06			
STEAP4	2.29	2.17E-07	ACTG2	-1.14	2.31E-03	IRF1	1.08	2.11E-06			
ETS2	1.28	2.22E-07	CCDC66	-1.03	2.91E-03	CD180	1.03	2.68E-06			
SLC2A3	1.50	2.44E-07	AQP5	-1.24	4.15E-03	LDLR	1.41	2.97E-06			
CXCR2	1.27	2.47E-07	GPAM	-1.16	5.74E-03	WARS	1.46	3.81E-06			
CYR61	1.94	2.61E-07	FABP7	-1.48	7.84E-03	FCGR2A	1.67	3.81E-06			

MARS	1.12	2.62E-07	SCGB1D1	-1.11	7.99E-03	UAP1	1.97	4.01E-06			
DNAJC2	1.09	2.70E-07	MUCL1	-1.60	9.80E-03	SLC1A3	1.19	4.06E-06			
IRF1	1.25	3.05E-07	SLC14A1	-1.12	1.06E-02	SOD2	1.73	4.33E-06			
MMP19	1.03	3.05E-07	SCGB1D2	-1.54	1.13E-02	CCR1	1.17	4.88E-06			
RRS1	1.16	3.45E-07	SLC12A2	-1.08	1.18E-02	DUSP6	1.14	5.25E-06			
CXCL1	1.45	3.84E-07	ACTA1	-1.10	1.37E-02	ADAMTS4	2.11	5.88E-06			
HAS2	1.61	3.94E-07	SERHL2	-1.07	1.78E-02	SRM	1.14	6.08E-06			
HSPH1	1.37	4.37E-07	SCGB2A2	-1.26	2.96E-02	PTGS2	1.26	8.99E-06			
SRGN	2.42	4.41E-07	PIP	-1.67	3.27E-02	TNFRSF12A	1.18	9.26E-06			
LCP1	1.54	4.46E-07				EGR1	1.58	1.01E-05			
SLC25A25	1.25	5.08E-07				CCL8	1.10	1.02E-05			
TUBA1C	1.14	5.79E-07				SRGN	1.90	1.02E-05			
FCGR2A	1.95	5.86E-07				PLSCR1	1.16	1.07E-05			
ADAMTS1	1.52	5.93E-07				THBD	1.17	1.07E-05			
PI3	1.69	6.11E-07				SLC5A6	1.17	1.11E-05			

MT1JP	1.07	6.28E-07				TNFAIP6	2.86	1.54E-05			
FGFBP1	1.79	6.54E-07				HAS2	1.34	1.67E-05			
ADAMTS9	1.72	6.92E-07				FOSL1	1.16	1.68E-05			
SPRR2A	2.95	7.32E-07				SPRED1	1.23	2.00E-05			
SLA	1.16	7.89E-07				HBEGF	1.12	2.19E-05			
SAMSN1	1.05	8.26E-07				ODC1	1.22	2.28E-05			
PLAUR	1.25	8.70E-07				ATP13A3	1.01	3.38E-05			
LCE2A	1.33	8.73E-07				DSG3	1.16	4.18E-05			
IL4R	1.22	8.86E-07				NR4A3	1.20	4.31E-05			
CTPS1	1.04	9.03E-07				TREM1	1.37	4.33E-05			
S100A12	2.03	9.18E-07				SLC7A5	1.29	4.36E-05			
IL36G	1.19	9.18E-07				FPR2	1.40	4.37E-05			
NOP2	1.04	9.72E-07				SECTM1	1.26	4.56E-05			
NCF2	1.66	1.06E-06				PI3	1.21	4.76E-05			
LCE1C	1.84	1.07E-06				CXCL2	1.11	5.00E-05			

CRISPLD2	1.30	1.07E-06				S100A12	1.50	6.43E-05			
GNA15	1.10	1.07E-06				SERPINE1	1.01	6.84E-05			
TIMP1	2.07	1.09E-06				ACSL4	1.06	1.02E-04			
SPRR2D	2.90	1.14E-06				HAS3	1.13	1.39E-04			
STEAP1	1.36	1.14E-06				DOK5	1.39	1.55E-04			
SPRR2E	2.67	1.30E-06				AQP3	1.07	1.62E-04			
SRM	1.27	1.35E-06				OSM	1.19	1.86E-04			
GALNT6	1.15	1.39E-06				NCF2	1.10	1.86E-04			
IL1R1	1.01	1.48E-06				CSRNP1	1.10	2.04E-04			
CD180	1.09	1.54E-06				SELL	1.34	2.07E-04			
CCL8	1.26	1.61E-06				PTX3	2.84	2.18E-04			
RAC2	1.24	1.62E-06				MMP1	1.01	2.19E-04			
GLIPR1	1.07	1.70E-06				KRT16	1.82	2.44E-04			
CSF3R	1.43	1.76E-06				SERPINE2	1.69	2.47E-04			
KRTDAP	1.23	2.05E-06				HIF1A	1.06	3.51E-04			

SPINK5	1.22	2.05E-06				IVL	1.25	3.66E-04			
FPR3	1.61	2.21E-06				FGFBP1	1.09	3.81E-04			
TMEM2	1.12	2.25E-06				B4GALT5	1.05	4.91E-04			
LCE2B	1.82	2.40E-06				KRT6A	2.14	6.28E-04			
PLSCR1	1.30	2.54E-06				GZMB	1.04	7.06E-04			
PNP	1.53	2.56E-06				PLAU	1.14	8.54E-04			
SAMHD1	1.04	2.65E-06				SLC7A11	1.00	9.16E-04			
FLG2	1.20	2.67E-06				AQP9	1.20	1.14E-03			
ASPRV1	2.04	2.87E-06				ATF3	1.22	1.14E-03			
NR4A3	1.51	2.93E-06				SPRR2A	1.38	1.14E-03			
IL6	2.51	3.12E-06				CCL19	1.12	1.29E-03			
AQP3	1.34	3.36E-06				S100A2	1.09	1.56E-03			
SERPINB4	2.52	3.43E-06				FOSB	1.95	1.79E-03			
BIRC3	1.16	3.90E-06				S100A7	1.49	3.33E-03			
S100A7	2.68	4.06E-06				SERPINB4	1.44	4.03E-03			

SERPINE1	1.23	4.81E-06				VNN2	1.16	4.82E-03			
SECTM1	1.48	4.84E-06				SPRR2D	1.41	5.05E-03			
SPRED1	1.35	4.89E-06				CLC	1.12	1.04E-02			
FFAR2	1.05	5.08E-06				SPRR1B	1.22	1.17E-02			
ALOX5AP	1.41	6.00E-06				FOS	1.56	1.50E-02			
PIM1	1.10	6.12E-06				CHI3L2	1.06	1.94E-02			
SELL	1.72	6.18E-06				SAA2	1.53	2.17E-02			
KPRP	1.15	6.24E-06				SPRR2B	1.01	2.87E-02			
GFPT2	1.59	6.35E-06				LRG1	1.06	3.82E-02			
FGL2	1.40	6.72E-06				KRT6B	1.07	4.31E-02			
LDLR	1.37	7.52E-06									
IVL	1.86	7.55E-06									
SBSN	1.05	7.71E-06									
THBD	1.16	7.85E-06									
AQP9	2.00	7.99E-06									

DUSP5	1.02	8.62E-06									
LINC00302	1.50	8.67E-06									
TNFRSF12A	1.20	9.08E-06									
ITGAM	1.31	9.18E-06									
ADAMTS4	2.09	9.40E-06									
FCER1G	1.58	9.82E-06									
SOD2	1.70	1.06E-05									
NFE2	1.51	1.09E-05									
CD1A	1.50	1.12E-05									
APOL1	1.12	1.16E-05									
MS4A6A	1.28	1.18E-05									
LCE2D	1.52	1.21E-05									
SPRR1B	2.16	1.31E-05									
TREM1	1.50	1.38E-05									
WARS	1.35	1.40E-05									

IL1RL1	1.06	1.41E-05									
PTGS2	1.26	1.44E-05									
FOSL1	1.30	1.47E-05									
FCN1	1.77	1.48E-05									
CTSC	1.05	1.48E-05									
SLC1A3	1.10	1.67E-05									
DMKN	1.09	1.68E-05									
TUBB2A	1.18	1.74E-05									
CLEC4G	1.43	1.90E-05									
SLC5A1	1.02	1.90E-05									
LCE1B	1.39	2.08E-05									
TYROBP	1.37	2.13E-05									
DOK2	1.03	2.17E-05									
ODC1	1.24	2.20E-05									
MNDA	1.05	2.20E-05									

ATF3	1.43	2.33E-05									
CA2	1.46	2.51E-05									
CRCT1	1.81	2.64E-05									
C1orf162	1.38	2.83E-05									
DOK5	1.60	2.86E-05									
DUSP6	1.03	3.17E-05									
FCGR3A	1.26	3.25E-05									
BATF3	1.14	3.32E-05									
LOR	2.17	3.33E-05									
LCE3D	2.09	3.39E-05									
LAPTM5	1.35	3.42E-05									
AKR1B10	1.21	3.49E-05									
HBEGF	1.09	3.71E-05									
CXCL2	1.16	3.77E-05									
CCL19	1.53	3.83E-05									

TLR4	1.42	3.91E-05									
SPRR2G	1.97	4.15E-05									
CMSS1	1.00	4.27E-05									
FPR2	1.44	4.28E-05									
CSRNP1	1.12	4.28E-05									
FLG	1.45	4.69E-05									
HAS3	1.24	5.08E-05									
GZMB	1.31	5.17E-05									
ANPEP	1.28	5.20E-05									
ALOX12B	1.00	5.28E-05									
TGM3	1.01	5.39E-05									
GSDMA	1.08	6.10E-05									
UGDH	1.24	6.37E-05									
SLURP1	1.36	6.60E-05									
C1orf68	1.19	6.60E-05									

OSM	1.33	7.01E-05									
FCGR2B	1.13	7.23E-05									
CD97	1.05	7.53E-05									
SERPINE2	1.88	7.89E-05									
ACSL4	1.08	8.31E-05									
HDC	1.36	9.00E-05									
MMP1	1.07	1.00E-04									
DSC1	1.04	1.00E-04									
PTX3	3.01	1.04E-04									
HTRA3	1.13	1.05E-04									
CST6	1.53	1.07E-04									
EGR1	1.40	1.10E-04									
MS4A4A	1.56	1.24E-04									
PDZK1IP1	1.11	1.28E-04									
SLC7A11	1.15	1.34E-04									

ANXA9	1.28	1.40E-04									
MEDAG	1.70	1.42E-04									
TNFRSF11B	1.13	1.51E-04									
PTGER2	1.11	1.60E-04									
LYNX1	1.31	1.67E-04									
LYN	1.03	1.72E-04									
SPP1	1.16	1.90E-04									
VNN2	1.63	2.05E-04									
MPEG1	1.20	2.08E-04									
LCE3B	1.83	2.10E-04									
CCR2	1.13	2.24E-04									
VCAN	1.49	2.36E-04									
CD14	1.35	2.66E-04									
RGS1	1.29	3.09E-04									
PRSS3	1.29	3.14E-04									

PLA2G2A	2.12	3.14E-04									
PSAPL1	1.37	3.18E-04									
MARCH3	1.24	3.32E-04									
LCE5A	1.30	3.45E-04									
CD300E	1.23	3.70E-04									
FOSB	2.29	3.91E-04									
C10orf99	1.75	4.97E-04									
CD163	1.79	5.89E-04									
EMB	1.07	7.04E-04									
SPRR2B	1.55	7.29E-04									
DAB2	1.10	9.56E-04									
CTSV	1.04	1.07E-03									
PTGIS	1.36	1.09E-03									
FOS	1.94	1.15E-03									
SMPD3	1.04	1.30E-03									

MARCO	1.28	1.99E-03									
IGFL2	1.20	2.77E-03									
FAP	1.34	3.10E-03									
KRT16	1.44	3.22E-03									
MATN4	1.25	3.32E-03									
KRT2	1.51	3.66E-03									
CHI3L2	1.29	3.78E-03									
CLC	1.28	4.67E-03									
COL6A6	1.06	5.00E-03									
DUSP1	1.02	5.69E-03									
CMA1	1.01	6.05E-03									
KRT6A	1.63	6.09E-03									
C3	1.10	6.22E-03									
CTSG	1.24	1.14E-02									
SAA2	1.71	1.23E-02									

C1QB	1.32	1.41E-02									
ATP12A	1.32	4.07E-02									
IGJ	1.06	4.16E-02									

Supplementary Table 2. Top 10 upregulated ToppGene pathways in CSU lesional skin versus healthy control skin.

Pathway	Q-Value	Genes Upregulated	Percentage* (%)
1. Keratinization	4.62E-56	*KRT6C, KRTAP5-8, LCE1C, LCE1B, LCE1E, LCE1A, LCE2D, LCE2C, LCE3B, LCE3A, LCE2A, LCE3E, KRTAP4-7, LCE5A, KRTAP4-9, KRT26, CDSN, KRTAP12-2, KRTAP12-1, KRT80, PI3, KLK5, KRT23, CSTA, KLK13, KRTAP5-5, KRTAP10-11, KRTAP10-4, KRTAP10-7, KRTAP10-12, LCE2B, KRTAP5-2, KRTAP5-7, KRTAP5-11, DSC1, KRTAP1-5, KRTAP3-2, KRTAP3-1, KRTAP9-8, KRTAP9-3, KRTAP17-1, KRTAP19-1, KRTAP19-5, KRTAP20-2, KRTAP5-4, KRTAP5-10, FLG, LCE6A, KRTAP9-6, SPRR1A, SPRR1B, SPRR2A, SPRR2B, SPRR2D, SPRR2E, SPRR2F, SPRR2G, KRTAP4-4, LCE3D, SPINK5, TGM1, KLK8, LCE4A, KRTAP4-8, KRTAP2-2, KRTAP9-1, KRTAP9-4, KRTAP4-1, KRTAP4-5, KRTAP4-2, KRTAP4-3, KRTAP3-3, KRTAP2-4, KRTAP9-7, IVL, RPTN, KRTAP26-1, KRT1, KRT2, KRT6A, KRT10, KRT16, KRT33A, KRT33B, KRT34, KRT83, KRT86, LORICRIN, KRTAP1-1, KRTAP1-3, KRTAP4-6, KRTAP9-9, KRTAP2-1*	43.5
2. Neutrophil degranulation	1.69E-23	*SERPINA3, ACP3, ADAM8, MMP9, MNDA, ALOX5, ANPEP, ARG1, CREG1, GGH, C3AR1, C5AR1, PNP, FCGR2C, CD14, CD33, CD53, CD68, ADGRE5, CTSC, SERPINA1, GCA, PLAU, PLAUR, GMFG, PRG2, CTSS, CYBB, PRSS2, PRSS3, CD55, PTAFR, CMTM6, PTPRC, PTX3, PYGL, CRISPLD2, DSC1, SIGLEC14, SERPINB1, PSMD14, PGM2, STOM, LILRB2, DOK3, FABP5, S100A7, S100A8, S100A9, S100A11, S100A12, TUBB4B, FCER1G, FCGR2A, FCGR3B, FCN1, SERPINB3, SELL, FPR1, FPR2, CCT2, SLC2A3, CD93,*	19.1

		SLC11A1, SLPI, CALML5, HPSE, FGL2, ADGRE3, GLIPR1, LILRB3, RAB31, ARHGAP9, NFAM1, CXCL1, MMP25, TLR2, TNFAIP6, MCEMP1, HLA-C, ABCA13, TYROBP, HSPA8, HSP90AA1, RETN, CXCR1, CXCR2, FLG2, ITGAM, ITGAX, ITGB2, KRT1, LAIR1, LYZ	
3. Formation of the cornified envelope	1.69E -23	*LCE1C, LCE1B, LCE1E, LCE1A, LCE2D, LCE2C, LCE3B, LCE3A, LCE2A, LCE3E, LCE5A, CDSN, PI3, KLK5, CSTA, KLK13, LCE2B, DSC1, FLG, LCE6A, SPRR1A, SPRR1B, SPRR2A, SPRR2B, SPRR2D, SPRR2E, SPRR2F, SPRR2G, LCE3D, SPINK5, TGM1, KLK8, LCE4A, IVL, RPTN, LORICRIN*	50.7
4. Innate Immune System	4.50E -17	*SERPINA3, ACP3, ADAM8, CCR2, MMP9, MNDA, ALOX5, ANPEP, BIRC3, ARG1, ATP6V1B2, NCF2, NCF4, CREG1, CFB, GGH, POLR3D, IER3, NFKBIB, C1QB, C3AR1, C5AR1, PNP, CASP4, FCGR2C, CD14, CD33, TREM1, CD53, CD68, ADGRE5, CDKN1A, CTSC, NLRC4, SERPINA1, PI3, PLA2G2A, GCA, PLAU, PLAUR, APBB1IP, MAP3K8, POLR1C, GMFG, PPP2CA, PRG2, MAPK13, MAP2K3, CTSL, CTSV, CTSS, CYBB, LGMN, PRSS2, PRSS3, CD55, PTAFR, DEFA3, CMTM6, DEFB4A, PTPRC, CLEC4A, PTX3, PYGL, CRISPLD2, DSC1, HBEGF, DUSP1, DUSP2, DUSP5, DUSP6, SIGLEC14, ARPC1B, ACTR3, SERPINB1, SPRED1, PSMD14, PGM2, STOM, EREG, LILRB2, NCF1, DOK3, CD209, FABP5, S100A7, S100A8, S100A9, S100A11, S100A12, TUBB4B, FCER1G, FCGR2A, FCGR3A, FCGR3B, FCN1, SERPINB3, FGF7, SELL, FOS, FPR1, FPR2, CCT2, SLC2A3, POLR3G, CD93, S100A7A, SLC11A1, SLPI, CD300E, DEFB103B, CALML5, HPSE, FGL2, NOD2, RNASE7, ADGRE3, GLIPR1, LILRB3, RAB31, ARHGAP9, NFAM1, CXCL1, MMP25, TLR2, TLR4, TNFAIP3, TNFAIP6, HCK, MCEMP1, HLA-C, ABCA13, TYROBP, HSPA8, HSP90AA1, LAT2, IFI16, RETN, IL1B, CXCR1, CXCR2, IRF1, PANX1, FLG2, ITGAM, ITGAX, ITGB2, ITGB3, MAPKAPK3, KRT1, LAIR1, LCP2, CD180, LYN, LYZ*	11.8
5. Developmental Biology	1.60E -14	*H2AC6, KRT6C, MMP9, MSN, KRTAP5-8, NOTCH1, CACNB4, RPS6KA4, DOK2, LCE1C, LCE1B, LCE1E, LCE1A, LCE2D, LCE2C, LCE3B, LCE3A, LCE2A, LCE3E, KRTAP4-7, LCE5A, KRTAP4-9, KRT26, CDSN, CEBPD, KRTAP12-2, KRTAP12-1, KRT80, PI3, CLTB, KLK5, APBB1IP, COL4A4, KRT23, PPP2CA, CSTA, KLK13, KRTAP5-5, KRTAP10-11, KRTAP10-4, KRTAP10-7, KRTAP10-12, LCE2B, PTPRC,*	12.0

		MAFB, KRTAP5-2, KRTAP5-7, KRTAP5-11, RAC2, DSC1, HBEGF, DUSP1, DUSP2, DUSP5, DUSP6, ARPC1B, ACTR3, EGR2, KRTAP1-5, KRTAP3-2, KRTAP3-1, KRTAP9-8, KRTAP9-3, KRTAP17-1, SPRED1, KRTAP19-1, PSMD14, EREG, KRTAP19-5, KRTAP20-2, COL6A5, KRTAP5-4, KRTAP5-10, FGF7, FLG, MED8, LCE6A, DOK5, KRTAP9-6, SPRR1A, SPRR1B, SPRR2A, SPRR2B, SPRR2D, SPRR2E, SPRR2F, SPRR2G, STAT3, KRTAP4-4, LCE3D, SPINK5, TGM1, KLK8, TNF, TYROBP, LCE4A, KRTAP4-8, KRTAP2-2, HSPA8, KRTAP9-1, HSP90AA1, KRTAP9-4, KRTAP4-1, KRTAP4-5, KRTAP4-2, KRTAP4-3, KRTAP3-3, KRTAP2-4, KRTAP9-7, ITGB3, IVL, RPTN, KRTAP26-1, KRT1, KRT2, KRT6A, KRT10, KRT16, KRT33A, KRT33B, KRT34, KRT83, KRT86, LORICRIN, KRTAP1-1, KRTAP1-3, KRTAP4-6, KRTAP9-9, KRTAP2-1, LYN	
6. Cytokine Signaling in Immune system	1.82E -14	*SEH1L, MCL1, CCR2, MMP1, MMP9, ALOX5, ANXA1, BIRC3, CCL3L3, MT2A, MX2, MYC, TNFSF14, IL18RAP, GBP5, SOCS3, NUP98, TNFRSF11B, OSM, IL1RL1, OSMR, CDKN1A, CEBPD, PIM1, CCR1, CCR5, APBB1IP, MAP3K8, SAMHD1, PPP2CA, CSF1R, CSF3, CSF3R, IL20, EIF4A3, PTAFR, IL22RA1, PTGS2, HBEGF, DUSP1, DUSP2, DUSP5, DUSP6, TRIM10, EGR1, EIF4A1, SPRED1, PSMD14, EREG, TNFRSF12A, FCGR1B, IFITM3, IFI30, CCL2, CCL3, FGF7, CCL4, CCL19, BATF, FOS, FPR1, IFITM2, IL36B, IL37, GBP1, STAT3, NOD2, CXCL1, CXCL2, TIMP1, TNF, IL36G, HCKHCK, IL34, HIF1A, HLA-C, HLA-DQA2, HLA-DQB1, HLA-DQB2, HMOX1, HSPA8, VCAM1, HSP90AA1, ICAM1, IFNAR2, IL1B, IL1R1, IL4R, IL6, IL7R, IL10, IL18, IRF1, IRF4, ISG20, ITGAM, ITGAX, ITGB2, ITGB3, JUNB, KPNA2, LTB, LYN*	13.5
7. Interleukin-10 signaling	8.42E -14	*CCR2, CCL3L3, CCR1, CCR5, CSF3, PTAFR, PTGS2, CCL2, CCL3, CCL4, CCL19, FPR1, STAT3, CXCL1, CXCL2, TIMP1, TNF, ICAM1, IL1B, IL1R1, IL6, IL10, IL18*	46.9
8. Interleukin-4 and 13 signaling	1.03E -13	*MCL1, MMP1, MMP9, ALOX5, ANXA1, MYC, SOCS3, OSM, CDKN1A, CEBPD, PIM1, PTGS2, CCL2, BATF, FOS, STAT3, TIMP1, TNF, HIF1A, HMOX1, HSPA8, VCAM1, HSP90AA1, ICAM1, IL1B, IL4R, IL6, IL10, IL18, IRF4, ITGAM, ITGAX, ITGB2, JUNB*	29.8
9. Staphylococcu	2.85E -11	*CFB, C1QB, C3AR1, C5AR1, FCGR2C, PTAFR, FCGR2A, FCGR2B, FCGR3A, FCGR3B, SELP, SELPLG, FPR1, FPR2,*	39.3

s aureus infection		*FPR3, HLA-DQA2, HLA-DQB1, ICAM1, IL10, ITGAM, ITGB2, KRT10*	
10. Signaling by Interleukins	3.05E-11	*MCL1, CCR2, MMP1, MMP9, ALOX5, ANXA1, CCL3L3, MYC, IL18RAP, SOCS3, OSM, IL1RL1, OSMR, CDKN1A, CEBPD, PIM1, CCR1, CCR5, APBB1IP, MAP3K8, PPP2CA, CSF1R, CSF3, CSF3R, IL20, PTAFR, IL22RA1, PTGS2, HBEGF, DUSP1, DUSP2, DUSP5, DUSP6, SPRED1, PSMD14, EREG, CCL2, CCL3, FGF7, CCL4, CCL19, BATF, FOS, FPR1, IL36B, IL37, STAT3, NOD2, CXCL1, CXCL2, TIMP1, TNF, IL36G, HCKHCK, IL34, HIF1A, HMOX1, HSPA8, VCAM1, HSP90AA1, ICAM1, IL1B, IL1R1, IL4R, IL6, IL7R, IL10, IL18, IRF4, ITGAM, ITGAX, ITGB2, ITGB3, JUNB, LYN*	14.1

*****Percentage of genes in the named pathway that were found to be upregulated in the dataset.

Supplementary Table 3. Selected upregulated ToppGene and KEGG pathways in CSU lesional skin versus healthy control skin.

**Pathway**	**Method**	**Q-Value**	**Genes Upregulated**	**Percentage* (%)**
IL-6 Mediated Signaling Events	Topp Gene	8.28E-4	*MCL1, MYC, SOCS3, CEBPD, FOS, STAT3, TIMP1, HCKHCK, IL6, IRF1, JUNB*	25.6
IL-12 Mediated Signaling Events	Topp Gene	3.80E-3	*GADD45B, IL18RAP, CCR5, MAP2K3, CCL3, CCL4, FOS, STAT3, GZMB, IL1B, IL1R1, IL18*	19.7
IL-18 Signaling	Topp Gene	2.05E-7	*MMP1, MMP9, BIRC3, FAS, ARL4D, ARG1, ATF3, NCF2, IL18RAP, IER3, SOCS3, CXCL16, CLDN1, TNFRSF11B, CYCS, CD83, CLDN4, PTGS2, PTX3, RGS16, NCF1, CCL2, CCL3, CCL4, CCL18, CCL19, ZC3H12A, FOS, SPP1, IL37, CXCL2, TIMP1, TNF, TNFAIP3, HMOX1, HSPB1, ICAM1, IL1B, IL6, IL10, IL18, IRF1, LTB*	15.4
IL-23 Mediated Signaling Events	Topp Gene	1.04E-3	*ALOX12B, IL18RAP, SOCS3, CCL2, STAT3, CXCL1, TNF, IL1B, IL6, IL18*	27.0

IL-17 Signaling	Topp Gene	3.60E-6	*MMP1, MMP9, CSF3, MAPK6, MAPK13, PTGS2, DEFB4A, S100A7, S100A8, S100A9, CCL2, FOS, FOSB, CXCL1, CXCL2, TNF, TNFAIP3, HSP90AA1, IL1B, IL6, FOSL1*	22.6
IL-17 Signaling	KEGG	3.75E-8	*CSF3, HSP90AA1, MMP1, TNFAIP3, CXCL1, FOS, PTGS2, CXCL2, TNF, MMP9, MAPK13, S100A7A, FOSL1, IL6, IL1B, CCL2, FOSB, DEFB4A, MAPK6, S100A9, S100A8, S100A7*	23.4
Th17 Differentiation	KEGG	0.017	*HSP90AA1, IL4R, IL1R1, STAT3, FOS, HIF1A, MAPK13, IL6, IRF4, IL1B, HLA-DQA2, NFKBIB, HLA-DQB1*	12.1

*****Percentage of genes in the named pathway that were found to be upregulated in the dataset.

Supplementary Table 4. Top 10 upregulated KEGG pathways in CSU lesional skin versus healthy control skin.

Pathway	Q-Value	Genes Upregulated	Percentage* (%)
1. Staphylococcus aureus infection	4.42 E-15	*C1QB, DEFB103B, ITGAM, SELPLG, ITGB2, C5AR1, PTAFR, FPR1, KRT23, FPR3, FPR2, ICAM1, KRT33B, KRT33A, FCGR3A, FCGR3B, KRT26, C3AR1, HLA-DQA2, IL10, KRT34, DEFA3, KRT10, SELP, FCGR2A, KRT16, DEFB4A, FCGR2B, FCGR2C, CFB, HLA-DQB1*	32.6
2. Viral protein interaction with cytokine and cytokine receptor	1.16E-14	*CSF1R, CCL13, IL20, CCL3L3, CXCR4, CXCL1, TNF, CXCL2, IL18RAP, CCL8, CXCR1, CCL4, CXCR2, CCL3, CCL2, CCR7, CCL19, CCR5, CCL18, CCR3, CCR2, CCR1, IL10, TNFSF14, IL37, IL34, TNFRSF10C, IL18, IL22RA1, IL6, CCL27*	31.0
3. Cytokine-cytokine receptor interaction	3.05E-12	*CSF3, IL20, CSF3R, CXCL1, TNF, CXCL2, CXCL16, IL18RAP, IL36B, CCR7, CCR5, CCR3, CCR2, IL10, IFNAR2, IL4R, TNFRSF12A, IL1R1, IL18, OSMR, IL1B, LTB, CCL13, CSF1R, CCL3L3, CXCR4, TNFRSF11B, IL1RL1, CCL8, CXCR1, CXCR2, CCL4, CCL3, CCL2, IL36RN, CCL19, CCL18, CCR1, TNFSF14, GDF15, IL37,*	16.9

		IL34, OSM, TNFRSF10C, IL36G, IL22RA1, IL6, FAS, IL7R, CCL27	
4. Phagosome	2.62E-10	*ITGAM, NCF1, NCF2, ITGB3, NCF4, ITGB2, CORO1A, THBS1, CTSS, TUBB8, TUBA1C, FCGR3A, FCGR3B, CTSL, SEC61G, MRC1, CD14, HLA-DQA2, CYBB, HLA-C, TUBB4B, TUBA4A, MARCO, TUBB2A, FCGR2A, CD209, ATP6V1B2, FCGR2B, TLR4, FCGR2C, HLA-DQB1, TLR2*	21.1
5. Osteoclast differentiation	2.62E-10	*LILRA6, CSF1R, NCF1, NCF2, ITGB3, NCF4, TNFRSF11B, TNF, LILRA4, SOCS3, FCGR3A, FCGR3B, JUNB, IFNAR2, IL1R1, LILRB1, FOS, LILRB2, LILRB3, LILRB4, MAPK13, FOSL1, TYROBP, FCGR2A, IL1B, FOSB, LCP2, FCGR2B, FCGR2C*	22.8
6. Lipid and atherosclerosis	5.12E-10	*CALML5, NCF1, NCF2, CCL3L3, NCF4, CXCL1, TNF, CXCL2, ICAM1, HSPD1, CCL3, CCL2, CD14, LDLR, LYN, MAP2K3, CYP2J2, HSPA8, HSP90AA1, VCAM1, HSPA5, HSPA4, MMP1, STAT3, IL18, CYBB, FOS, SOD2, SELE, MMP9, MAPK13, SELP, IL6, IL1B, FAS, CYCS, TLR4, TLR2*	17.7
7. Leishmaniasis	4.76E-9	*IL10, ITGAM, NCF1, NCF2, ITGB2, NCF4, CYBB, FOS, PTGS2, TNF, MAPK13, FCGR3A, FCGR3B, FCGR2A, IL1B, TLR4, HLA-DQA2, FCGR2C, HLA-DQB1, TLR2, NFKBIB*	27.3
8. IL-17 signaling pathway	3.75E-8	*CSF3, HSP90AA1, MMP1, TNFAIP3, CXCL1, FOS, PTGS2, CXCL2, TNF, MMP9, MAPK13, S100A7A, FOSL1, IL6, IL1B, CCL2, FOSB, DEFB4A, MAPK6, S100A9, S100A8, S100A7*	23.4
9. TNF signaling pathway	4.02E-8	*MAP2K3, VCAM1, TNFAIP3, CXCL1, FOS, NOD2, PTGS2, TNF, SELE, CXCL2, MMP9, ICAM1, MAPK13, RPS6KA4, SOCS3, IL6, CREB3L4, IL1B, IRF1, CCL2, FAS, MAP3K8, JUNB, BIRC3*	21.4
10. Rheumatoid arthritis	1.43E-7	*MMP1, CCL3L3, ITGB2, IL18, CXCL1, FOS, CXCL2, TNF, ICAM1, IL6, CTSL, IL1B, ATP6V1B2, CCL3, CTLA4, CCL2, LTB, TLR4, HLA-DQA2, HLA-DQB1, TLR2*	22.6

*****Percentage of genes in the named pathway that were found to be upregulated in the dataset.

Supplementary Table 5. Top 10 upregulated ToppGene pathways in CSU lesional skin versus non-lesional skin.

Pathway	Q-Value	Genes Upregulated	Percentage * (%)
1. Interleukin-10 signaling	1.05E-14	*CCL2, CCL3, CCL4, CCL19, FPR1, ICAM1, CCL3L3, CSF3, IL1B, IL1R1, IL6, IL10, PTAFR, PTGS2, STAT3, CXCL1, CXCL2, TIMP1*	36.7
2. Cytokine Signaling in Immune system	1.70E-10	*CDKN1A, SEH1L, HIF1A, TNFRSF12A, FCGR1B, PIM1, IFI30, CCL2, CCL3, CCL4, MMP1, CCL19, HSPA8, VCAM1, FPR1, PIK3R3, ICAM1, CCL3L3, MT2A, CSF3, IL20, IL1B, IL1R1, IL4R, IL6, IL7R, MYC, IL10, EIF4A3, IRF1, PTAFR, ITGB3, PTGS2, STAT3, JUNB, NFKB1, KPNA2, HBEGF, DUSP2, DUSP5, DUSP6, SOCS3, CXCL1, CXCL2, OSM, TIMP1, EGR1, EIF4A1, SPRED1, IL1RL1, OSMR, LYN*	6.8
3. Signaling by Interleukins	2.60E-10	*CDKN1A, HIF1A, PIM1, CCL2, CCL3, CCL4, MMP1, CCL19, HSPA8, VCAM1, FPR1, PIK3R3, ICAM1, CCL3L3, CSF3, IL20, IL1B, IL1R1, IL4R, IL6, IL7R, MYC, IL10, PTAFR, ITGB3, PTGS2, STAT3, JUNB, NFKB1, HBEGF, DUSP2, DUSP5, DUSP6, SOCS3, CXCL1, CXCL2, OSM, TIMP1, SPRED1, IL1RL1, OSMR, LYN*	7.9
4. Neutrophil degranulation	1.38E-9	*SERPINA3, FABP5, S100A7, S100A8, S100A9, S100A12, SERPINA1, FCGR2A, FCGR3B, SERPINB3, GCA, PLAU, HSPA8, SELL, ANPEP, FPR1, FPR2, CCT2, SLC2A3, CD93, CXCR1, CXCR2, PTAFR, FGL2, PTPRC, PTX3, ADGRE3, NFKB1, C5AR1, PNP, CRISPLD2, LILRB3, CXCL1, FCGR2C, PA2G4, CD53, SERPINB1, TNFAIP6, STOM*	7.9
5. Interleukin-4 and 13 signaling	2.15E-9	*CDKN1A, HIF1A, PIM1, CCL2, MMP1, HSPA8, VCAM1, ICAM1, IL1B, IL4R, IL6, MYC, IL10, PTGS2, STAT3, JUNB, SOCS3, OSM, TIMP1*	16.7
6. AP-1 transcription factor network	7.68E-9	*HIF1A, CCL2, PLAU, MMP1, FOSB, MT2A, CCN1, ATF3, IL6, MYC, IL10, JUNB, FOSL1, TIMP1, EGR1*	21.4
7. rRNA modification in	1.84E-8	*UTP15, WDR36, NOP58, NOP14, PDCD11, UTP14A, WDR43, WDR3, DKC1, UTP20, NOP2, HEATR1, UTP4, NAT10*	22.2

the nucleus and cytosol			
8. Ribosome biogenesis in eukaryotes	3.43E-8	*GTPBP4, LSG1, UTP15, GNL2, XRN2, WDR36, NOP58, UTP14A, WDR43, WDR3, DKC1, GNL3, RAN, RIOK1, HEATR1, UTP4, NAT10*	16.0
9. Ensemble of genes encoding ECM-associated proteins including ECM-affiliated proteins, ECM regulators and secreted factors	1.43E-7	*SERPINA3, S100A2, S100A3, S100A7, S100A8, S100A9, S100A12, SERPINA1, PI3, SERPINE2, CLC, SERPINB13, SERPINB3, SERPINB4, CCL2, CCL3, CCL4, PLAU, CCL8, CCL13, MMP1, CCL19, MMP12, PLOD2, ADAMTS4, ADAMTS1, CCL3L3, AREG, CSF3, IL20, IL1B, CTSL, IL6, IL10, ADAMTS9, CLEC4A, FGFBP1, HBEGF, CXCL1, CXCL2, OSM, TIMP1, SERPINE1, SERPINB1, GREM1*	6.0
10. Lung fibrosis	1.61E-7	*SERPINA1, CCL2, CCL3, CCL4, PLAU, MT2A, CSF3, IL1B, IL6, PTX3, CXCL2, TIMP1, GREM1*	20.6

*****Percentage of genes in the named pathway that were found to be upregulated in the dataset.

Supplementary Table 6. Selected upregulated ToppGene and KEGG pathways in lesional versus non-lesional CSU samples.

Pathway	Method	Q-Value	Genes Upregulated	Percentage* (%)
IL-18 Signaling	ToppGene	1.72E-4	*CCL2, CCL3, CCL4, MMP1, CCL19, ZC3H12A, ICAM1, ATF3, IL1B, IL6, IL10, IRF1, NCF2, PTGS2, PTX3, NFKB1, SOCS3, CXCL2, RGS16, TIMP1*	7.2
IL-6 Mediated Signaling Events	ToppGene	1.28E-3	*IL6, MYC, IRF1, STAT3, JUNB, SOCS3, TIMP1*	16.3
IL-12 Mediated Signaling Events	ToppGene	2.57E-2	*CCL3, CCL4, IL1B, IL1R1, STAT3, NFKB1*	9.8

IL-17 Signaling	Topp Gene	1.04E-3	*S100A7, S100A8, S100A9, CCL2, MMP1, FOSB, CSF3, IL1B, IL6, PTGS2, NFKB1, CXCL1, CXCL2, FOSL1*	15.1
IL-23 Mediated Signaling Events	Topp Gene	5.59E-4	*CCL2, IL1B, IL6, STAT3, NFKB1, SOCS3, CXCL1*	18.9
IL-4 Mediated Signaling Events	Topp Gene	0.029	*SELP, IL4R, IL10, ITGB3, SOCS3, EGR2*	9.50
IL-17 Signaling	KEGG	9.20E-8	*CSF3, MMP1, CXCL1, PTGS2, CXCL2, NFKB1, FOSL1, IL6, IL1B, CCL2, FOSB, S100A9, S100A8, S100A7*	14.9
Th17 Differentiation	KEGG	0.019	*IL6, IL4R, IL1R1, IL1B, STAT3, HIF1A, NFKB1*	6.5

*****Percentage of genes in the named pathway that were found to be upregulated in the dataset.

Supplementary Table 7. Top 10 upregulated KEGG pathways in CSU lesional skin versus non-lesional skin.

**Pathway**	**Q-Value**	**Genes Upregulated**	**Percentage* (%)**
1. Lipid and atherosclerosis	1.36E-9	*LYN, HSPA8, VCAM1, HSPA5, NCF2, MMP1, CCL3L3, STAT3, PIK3R3, CXCL1, SOD2, EIF2S1, SELE, CXCL2, NFKB1, ICAM1, HSPD1, SELP, IL6, IL1B, CCL3, CCL2, LDLR*	10.7
2. Ribosome biogenesis in eukaryotes	1.36E-9	*UTP15, WDR36, NOP58, UTP4, WDR3, HEATR1, NAT10, GNL2, GTPBP4, WDR43, GNL3, LSG1, DKC1, XRN2, RIOK1, UTP14A, RAN*	15.7
3. Osteoclast differentiation	1.28E-8	*NCF2, IL1R1, ITGB3, PIK3R3, LILRB3, NFKB1, FOSL1, SOCS3, FCGR3A, FCGR3B, FCGR2A, IL1B, FOSB, LCP2, FCGR2B, JUNB, FCGR2C*	13.4

4. IL-17 signaling pathway	9.20E-8	*CSF3, MMP1, CXCL1, PTGS2, CXCL2, NFKB1, FOSL1, IL6, IL1B, CCL2, FOSB, S100A9, S100A8, S100A7*	15.2
5. TNF signaling pathway	9.20E-8	*VCAM1, PIK3R3, CXCL1, PTGS2, SELE, CXCL2, NFKB1, ICAM1, SOCS3, IL6, IL1B, IRF1, BCL3, CCL2, JUNB*	13.4
6. Viral protein interaction with cytokine and cytokine receptor	1.48E-7	*IL10, CCL13, IL20, CCL3L3, CXCL1, CXCL2, IL6, CCL8, CXCR1, CCL4, CXCR2, CCL3, CCL2, CCL19*	14.0
7. Cytokine-cytokine receptor interaction	1.92E-7	*IL10, CCL13, CSF3, IL20, IL4R, TNFRSF12A, IL1R1, CCL3L3, OSM, CXCL1, OSMR, CXCL2, IL1RL1, IL6, CCL8, CXCR1, IL1B, CXCR2, CCL4, CCL3, CCL2, CCL19, IL7R*	7.8
8. Staphylococcus aureus infection	5.06E-7	*IL10, C5AR1, PTAFR, FPR1, FPR2, ICAM1, SELP, FCGR3A, FCGR3B, FCGR2A, KRT16, FCGR2B, FCGR2C*	13.7
9. Malaria	5.06E-7	*IL10, SELP, CSF3, IL6, VCAM1, IL1B, CCL2, THBS1, SELE, ICAM1*	20.0
10. AGE-RAGE signaling pathway in diabetic complications	8.01E-7	*EGR1, VCAM1, SERPINE1, STAT3, PIK3R3, SELE, NFKB1, ICAM1, THBD, IL6, IL1B, PIM1, CCL2*	13.0

*****Percentage of genes in the named pathway that were found to be upregulated in the dataset.

Supplementary Table 8. Cell-types with significant differences in the Gimenez et al. dataset.

**Cell Type**	**Cell-Type Enrichment Methods**	**Lesional Skin Mean Score**	**Non-Lesional Skin Mean Score**	**Healthy Control Skin Mean Score**	**Q-Value Lesional vs. Non-lesional Skin**	**Q-Value Lesional vs. Healthy Control Skin**
Astrocytes	xCell	0.00093	-	0.032	-	0.028
Dendritic Cells	xCell	0.18	-	0.056	-	0.024

Endothelial Cells	xCell	0.14	-	0.25	-	0.028
Epithelial Cells	xCell	0.040	-	0.012	-	0.045
Keratinocytes	xCell	0.079	-	0.031	-	0.037
Megakaryocyte-Erythroid Progenitor Cells	xCell	0.064	-	0.0070	-	0.040
M1 Macrophages	xCell	0.046	-	0.0087	-	0.030
Mesangial cells	xCell	0.0067	-	0.072	-	0.0013
Monocytes	xCell	0.18	-	0.048	-	0.040
Neurons	xCell	0.019	-	0.034	-	0.048
Neutrophils	xCell	0.077	-	0.0038	-	0.037
Osteoblast	xCell	0.096	-	0.019	-	0.030
Pericytes	xCell	0.0047	-	0.019	-	0.030
Preadipocytes	xCell	0.11	-	0.017	-	0.028
Sebocytes	xCell	0.014	-	0.0039	-	0.0070
Tgd cells	xCell	0.091	-	0.015	-	0.028
Activated Dendritic Cells	xCell	0.13	-	0.039	-	0.026
Lymphatic Endothelial Cells	xCell	0.12	-	0.22	-	0.028
Microvascular Endothelial Cells	xCell	0.10	-	0.20	-	0.030
CD8+ T-Cells	CIBERSORT	0.020	-	0.15	-	0.049

Supplementary Table 9. Cell-types with significant differences in the Patel et al. dataset.

Cell Type	Cell-Type Enrichment Method	Lesional Skin Mean Score	Non-Lesional Skin Mean Score	Healthy Control Skin Mean Score	Q-Value Lesional vs. Non-lesional Skin	Q-Value Lesional vs. Healthy Control Skin
CD4+ Memory T-Cells	xCell	0.11	0.057	-	0.039	-
Naïve CD8+ T-Cells	xCell	0.0048	0.035	-	0.037	-
Lymphoid Progenitor Cells	xCell	0.031	0.0077	-	0.024	-
Common Myeloid Progenitor Cell	xCell	0.0072	0.059	-	0.019	-
Chondrocytes	xCell	0.018	0.071	-	0.024	-
Dendritic Cells	xCell	0.15	0.085	-	0.037	-
Hematopoietic Stem Cells	xCell	0.053	0.16	-	0.024	-
Hepatocytes	xCell	0	0.0094	-	0.024	-
Megakaryocyte-Erythroid Progenitor Cells	xCell	0.026	0.0014	-	0.039	-
Macrophages	xCell	0.024	0.051	-	0.039	-
Neurons	xCell	0.0085	0.020	-	0.049	-
Neutrophils	xCell	0.11	0.0094	-	0.024	-
Skeletal muscle	xCell	0.00037	0.011	-	0.024	-
Activated Dendritic Cells	xCell	0.096	0.051	-	0.019	-
Immature Dendritic Cells	xCell	0.16	0.093	-	0.039	-

Lymphatic Endothelial cells	xCell	0.099	0.045	-	0.039	-
Plasma Cells	CIBERSORT	0.018	0.042	0.057	0.032	0.015
CD8+ T-Cells	CIBERSORT	0.035	-	0.090	-	0.028
CD4+ Resting Memory T-Cells	CIBERSORT	0.13	0.038	0.037	0.032	0.035
Tregs	CIBERSORT	0.00094	0.030	0.034	0.032	0.015
Resting Mast Cells	CIBERSORT	0.14	0.27	0.25	0.029	0.016

Supplementary Table 10. Cell-types with significant differences between datasets.

Sample Type	Cell Type	Deconvolution Method	Patel et al. Mean Score	Gimenez et al. Mean Score	Q-Value
Lesional Skin	Naïve B-Cells	CIBERSORT	0.040	0.011	0.014
Lesional Skin	Resting Dendritic Cells	CIBERSORT	0.21	0.049	0.014
Non-Lesional Skin	CD4+ Central Memory T-Cells	xCell	0.017	0	0.029
Non-Lesional Skin	CD8+ T-Cells	xCell	0.018	0.072	0.044
Non-Lesional Skin	Lymphoid Progenitor Cell	xCell	0.0077	0.044	0.024
Non-Lesional Skin	Chrondrocytes	xCell	0.071	0.18	0.018
Non-Lesional Skin	Endothelial Cells	xCell	0.033	0.19	0.0080
Non-Lesional Skin	Granulocyte-Monocyte Progenitor Cells	xCell	0.012	0.089	0.044

Non-Lesional Skin	Hematopoietic Stem Cells	xCell	0.16	0.35	0.0080
Non-Lesional Skin	Monocytes	xCell	0.030	0.13	0.029
Non-Lesional Skin	Myocytes	xCell	0.0089	0.043	0.020
Non-Lesional Skin	Neurons	xCell	0.020	0.032	0.044
Non-Lesional Skin	Th1 Cells	xCell	0.054	0	0.016
Non-Lesional Skin	Th2 Cells	xCell	0	0.029	0.046
Non-Lesional Skin	Lymphatic Endothelial Cells	xCell	0.044	0.15	0.016
Non-Lesional Skin	Naïve B-Cells	CIBERSORT	0.050	0.0091	0.0058
Non-Lesional Skin	Activated NK Cells	CIBERSORT	0.070	0.0024	0.0011
Non-Lesional Skin	Monocytes	CIBERSORT	0.18	0.018	0.0011
Non-Lesional Skin	M2 Macrophages	CIBERSORT	0.010	0.28	0.011
Non-Lesional Skin	Resting Dendritic Cells	CIBERSORT	0.15	0.041	0.0058
Non-Lesional Skin	Resting Mast Cells	CIBERSORT	0.27	0.16	0.042
Healthy Control Skin	Adipocytes	xCell	0.21	0.074	0.046
Healthy Control Skin	Chondrocytes	xCell	0.025	0.12	0.014
Healthy Control Skin	Endothelial Cells	xCell	0.0080	0.25	0.0047

Healthy Control Skin	Epithelial Cells	xCell	0.074	0.012	0.0083
Healthy Control Skin	Fibroblasts	xCell	0.049	0.13	0.041
Healthy Control Skin	Granulocyte-Monocyte Progenitor Cells	xCell	0.0027	0.064	0.046
Healthy Control Skin	Hematopoietic Stem Cells	xCell	0.13	0.32	0.0047
Healthy Control Skin	Keratinocytes	xCell	0.071	0.031	0.046
Healthy Control Skin	Mesangial Cells	xCell	0.0092	0.072	0.0047
Healthy Control Skin	Monocytes	xCell	0.0038	0.048	0.041
Healthy Control Skin	Myocytes	xCell	0.0098	0.057	0.0047
Healthy Control Skin	Neurons	xCell	0.024	0.034	0.047
Healthy Control Skin	Sebocytes	xCell	0.0095	0.0039	0.046
Healthy Control Skin	Th1 Cells	xCell	0.074	0	0.0047
Healthy Control Skin	Lymphatic Endothelial Cells	xCell	0.020	0.22	0.0047
Healthy Control Skin	Microvascular Endothelial Cells	xCell	0.047	0.20	0.0057
Healthy Control Skin	Naïve B-Cells	CIBERSORT	0.058	0	0.019
Healthy Control Skin	Activated NK Cells	CIBERSORT	0.073	0.014	0.0068

Healthy Control Skin	Monocytes	CIBERSORT	0.21	0.017	0.0069
Healthy Control Skin	M2 Macrophages	CIBERSORT	0.067	0.30	0.030
Healthy Control Skin	Resting Dendritic Cells	CIBERSORT	0.12	0.012	0.019
